# Six new species of *Begonia* (Begoniaceae) from limestone areas in Northern Vietnam

**DOI:** 10.1186/s40529-015-0089-3

**Published:** 2015-05-02

**Authors:** Ching-I Peng, Che-Wei Lin, Hsun-An Yang, Yoshiko Kono, Hieu Quang Nguyen

**Affiliations:** 1 Herbarium (HAST), Biodiversity Researach Center, Academia Sinica, Taipei 115, Nangang, Taiwan; 2 Division of Botanical Garden, Taiwan Forestry Research Institute, No. 53, Nan–Hai Road, Taipei, 10066 Taiwan; 3 Center for Plant Conservation (CPC), Vietnam Union of Science and Technology Associations No. 25/32, Lane 191, Lac Long Quan Road, Nghia Do, Hanoi, Cau Giay District, Vietnam

**Keywords:** Begonia caobangensis, Begonia circularis, Begonia melanobullata, Begonia langsonensis, Begonia locii, Begonia montaniformis, Limestone, New species, sect. Coelocentrum, sect. Platycentrum,Vietnam

## Abstract

**Background:**

Species of *Begonia* are richly represented in limestone karst areas across the Sino-Vietnamese border. More than one hundred species were known, many of which were documented recently.

**Results:**

In continuation of our systematic studies of Asian *Begonia*, we report six species of *Begonia* that are unknown to science, namely *B. caobangensis* [sect. *Platycentrum*]*, B. circularis, B. melanobullata, B. langsonensis, B.locii* and *B. montaniformis* [sect. *Coelocentrum*] from Northern Vietnam. Diagnostic features that separate them from morphologically allied species are provided. Somatic chromosome numbers were determined, which supports their placement in the respective sections. Foliar SEM microphotographs were taken and described.

**Conclusion:**

A careful study of the literature, herbarium specimens and living plants, both in the wild and in cultivation in the experimental greenhouse, supports the recognition of the six new species, which are described and illustrated.

**Electronic supplementary material:**

The online version of this article (doi:10.1186/s40529-015-0089-3) contains supplementary material, which is available to authorized users.

## Background

*Begonia* sect. *Coelocentrum* is richly represented in limestone karst areas across the Sino-Vietnamese border region, comprising more than 60 species (Peng et al. [2014a], Chung et al. [2014]). Nearly half of the species in sect. *Coelocentrum* were discovered in the past decade (Chung et al., [2014]; Peng et al. [2013], [2014a]), mostly from southern China. The area from South China to North Vietnam harbors very high levels of biodiversity (Sodhi et al. [2004]). Recently, Averyanov and Nguyen ([2012]) documented ten new species of *Begonia* from Vietnam and one new species from Laos. They also estimated the total actual number of *Begonia* species in eastern Indochina to be 180–200 species. It seems reasonable to speculate that there are more species of *Begonia* sect. *Coelocentrum* to be discovered in the future. In this study we report five new species of sect. *Coelocentrum* from Vietnam, some with very handsome maculation pattern and/or attractive leaf texture and an unusual new species of sect. *Platycentrum* with lanceolate and symmetric leaves. Detailed morphological descriptions, line drawings, color plates and foliar SEM microphotographs are provided, and chromosome numbers are reported.

## Methods

### Chromosome preparations

Somatic chromosomes were studied for plants of *Begonia caobangensis* (*Peng 23895*, HAST), *B. circularis* (*Peng 22610*, HAST), *B. melanobullata* (*Peng 22609,* HAST), *B. langsonensis* (*Peng 21946*, HAST), *B. locii* (*Peng 21943*, HAST) and *B. montaniformis* (*Peng 24609, 24610, 24613,* HAST) using root tips. The procedures of pretreatment, fixation, and staining for chromosome observations were described in our previous paper (Peng et al. [2014b]). Classification of the chromosome complements based on centromere position at mitotic metaphase followed Levan et al. ([1964]). Voucher specimens of all new species were deposited in the Herbarium of the Biodiversity Research Center, Academia Sinica (HAST).

### Cryo scanning electron microscopy

Fresh leaves of *Begonia caobangensis*, *B. circularis*, *B. melanobullata*, *B. langsonensis*, *B. locii* and *B. montaniformis* were dissected and attached to a stub. The samples were frozen with liquid nitrogen slush, then transferred to a sample preparation chamber at −160°C and etched for 15 min at −85°C. After etching, the temperature reached −130°C for sample fracturing and coating. After coating, the samples were transferred to the SEM chamber and observed at −190°C with a cryo scanning electron microscope (FEI Quanta 200 SEM/Quorum Cryo System PP2000TR FEI).

## Results and discussion

### Species description

**Figure 1:**
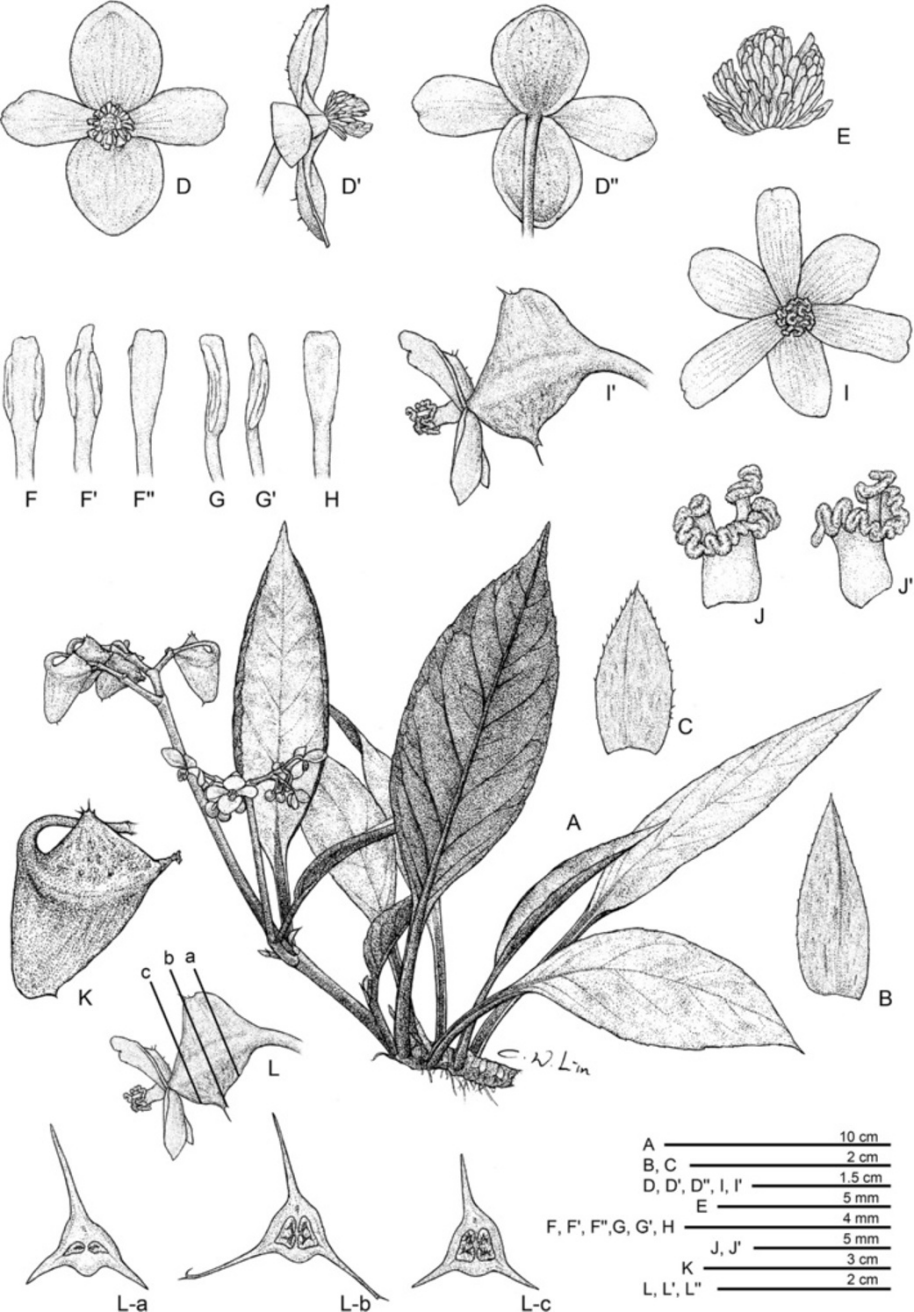
*Begonia caobangensis* C.-I Peng & C. W. Lin. **A**. Habit; **B**. Stipule, abaxial view; **C**. Bract; **D**, **D**’, **D**”. Staminate flowers; **E**. Androecium; **F**, **G**, **H**. Stamens; **I**, **I**’. Pistillate flowers; **J**, **J**’. Style and stigmatic band; **K**. Capsule; **L**. Cross section of an immature capsule. All from *Peng 23895* (HAST).

**Figure 2:**
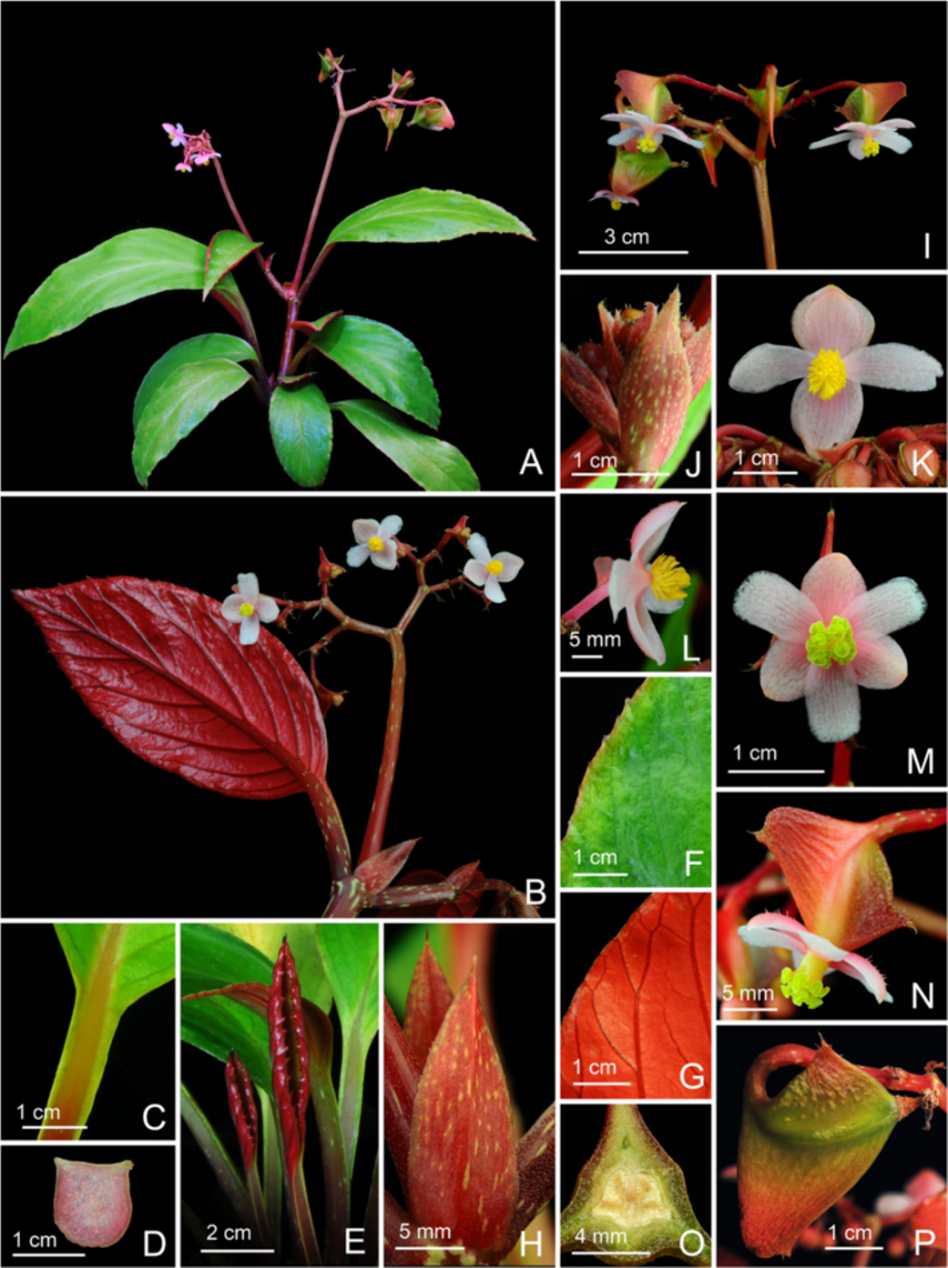
*Begonia caobangensis* C.-I Peng & C. W. Lin*.* **A**. Habit; **B**. Blade, abaxial view with an inflorescence; **C**. Winged petiole; **D**. Cross section of petiole; **E**. Infolded juvenile leaves; **F**, **G**. Portion of leaf, adaxial and abaxial surface; **H**. Stipules on rhizome; **I**. Inflorescence, showing pistillate flowers; **J**. Bracts; **K**, **L**. Staminate flowers; **M**, **N**. Pistillate flowers; **O**. Cross section of an immature capsule; **P**. Capsule. All from *Peng 23895* (HAST).

**1.** ***Begonia caobangensis*** C.-I Peng & C. W. Lin, sp. nov. (sect. *Platycentrum*) **—TYPE:** VIETNAM. Cao Bang Province, Tra Linh District, Thang Hen Lake, elev. ca. 1000 m. Growing as solitary plants on soil slope of 30° in forest with light to medium shade, plant not situated in proximity to the water but in drier area up the bank. Originally collected by Ms. Mary Sizemore on 19 May 2005 and distributed to American Begonia Society as *Begonia* U555. Type specimens pressed from plants cultivated in the experimental greenhouse in Academia Sinica, Taiwan, 9 June 2014, *Peng 23895* (holotype: HAST; isotypes: A, E, HN) (Figures 1 and 2).

Herbs, monoecious, perennial, rhizomatous. **Rhizome** creeping, 1–2 cm thick, internodes 0.2–0.5 cm long, minutely appressed tomentose. **Stipules** deciduous, reddish, ovate, 2–2.5 cm long, 0.7–1 cm wide, keeled, abaxially minutely appressed tomentose, sometimes puberulous along midrib, margin denticulate, apex aristate, arista ca. 1 mm long. **Petioles** reddish to crimson, flat front, rounded back (D-shaped in cross section), 3.5–8 (−10) cm long, 0.5–0.7 cm thick, minutely appressed tomentose to glabrous, narrowly winged on both sides, wings 1–2.5 mm wide. **Leaves** alternate, adaxially lime green, abaxially reddish to crimson, juvenile blade folding inward, mature blade flat, symmetric, narrowly elliptic to elliptic or slightly rhomboid, (8−) 11–21.5 cm long, 3.9–7.3 cm wide, apex acuminate, base attenuate, margin repand to sparsely denticulate, chartaceous to thinly coriaceous, glabrous (minutely appressed tomentose on young leaves), venation 6–8-pinnate on each side of the midrib, secondary veins branching dichotomously or nearly so, tertiary veins weakly percurrent. **Inflorescences** terminal or axillary on upper nodes, cymes dichasial, branched 3–4 times; peduncle 8–12 cm long, tomentose or subglabrous; bracts and bracteoles caducous, reddish, bracts ovate, ca. 1.8 cm long, 0.6–0.9 cm wide, boat-shaped, veins crimson, margin denticulate or ciliate, bracteoles similar but smaller. **Staminate flower:** pedicel 1.3–2.1 cm long, tepals 4, outer 2 broadly ovate, 12–18 mm long, 10–17 mm wide, abaxially reddish, sparsely setulose, inner 2 pinkish, obovate, 13–16 mm long, 6–8 mm wide; androecium actinomorphic, spheric, 6–8 mm across; stamens 85–120; anthers yellow, slightly compressed, lanceolate to oblanceolate, apex obtuse to retuse, ca. 2.5 mm long; filaments shorter than anthers, slightly fused at base. **Pistillate flower:** pedicel 1.2–1.8 cm long, tepals 6, pinkish-white, outer 3 obovate to elliptic, 8–14 mm long, 6–9 mm wide, inner 3 oblong to narrowly elliptic,9–18 mm long, 4–6.5 mm wide; ovary reddish, trigonous-ellipsoid, 12–15 mm long, 4–5 mm thick (wings excluded), 3-winged, wings unequal, reddish-yellow, each tipped with several succulent bristles 2–4 mm long; the 2 lateral wings 5.5–8 mm wide, shallowly triangular, verrucose, abaxial wing crescent-shaped, 10–13 mm wide; styles 2, fused at base, yellow, ca. 5 mm long, stigma strongly undulate and spirally twisted. **Capsule** trigonous-ellipsoid, 18–25 mm long, 4–7 mm thick (wings excluded), greenish or reddish when fresh; wings unequal, lateral wings 7–15 mm wide, abaxial wing crescent-shaped, 20–25 mm wide. Somatic chromosome number, 2*n* = 22.

#### Distribution and ecology

**Figure 3:**
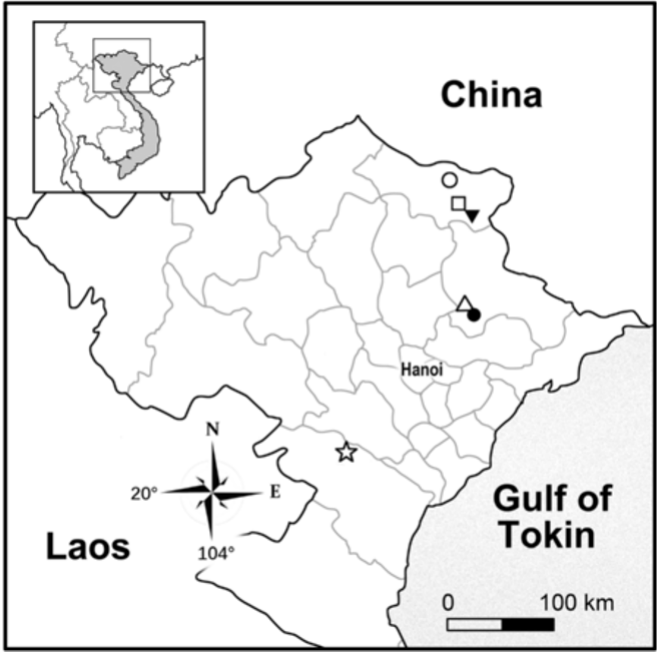
Distribution map of *Begonia caobangensis* (open circle), *B. circularis* (Inverted solid triangle), *B. langsonensis* (open triangle), *B. locii* (solid circle), *B. melanobullata* (square) and *B. montaniformis* (star) in Northern Vietnam.

Northern Vietnam, around Thang Hen Lake, Tra Linh District, Cao Bang Province (Figure 3). Situated among limestone mountains, near the border of Vietnam and China. Growing on soil slope in evergreen broad-leaved forest, in light shade, at ca.1,000 m in elevation (Mary Sizemore, pers. comm.). *Begonia caobangensis* occurs also in Tuyen Quang province (Trinh Ngoc Bon, pers. comm.), which is adjacent to the southwest border of Cao Bang.

#### Etymology

The specific epithet refers to the locality of the type collection.

#### Notes

*Begonia caobangensis* resembles *B. aequilateralis* Irmsch., also a member of sect. *Platycentrum*, in having symmetric and elliptic leaves. However, *B. caobangensis* is markedly distinct in many other features such as blade base decurrent (vs. obtuse to cuneate), narrowly winged (vs. not winged) petiole, presence (vs. lacking) of the succulent bristles at tips of ovary wings and pistillate tepals 6 (vs. 5). A comparison of the two species is presented in Table 1. The combination of symmetric leaves, 6-tepalled pistillate flowers and the succulent bristles at tips of ovary wings make it a unique species in sect. *Platycentrum*.

**Table 1:** **Comparison of** ***Begonia caobangensis*** **and** ***B. aequilateralis***

	***B. caobangensis***	***B. aequilateralis***
**Stipule**		
Size (cm)	2–2.5 × 0.7–1	1 × 0.3
**Petiole**	Narrowly winged	Not winged
**Leaf**		
Base	Decurrent	Obtuse to cuneate
**Staminate flower**		
Tepal size (mm)	Outer 10–12 × 4–8, inner 7–15 × 2–6	Outer 12–18 × 10–17, inner 13–16 × 6–8
**Pistillate flower**		
Tepal number	6	5
Tepal size (mm)	Outer 8–14 × 6–9, inner 9–18 × 4–6.5	Outer 13–17 × 5–7.5, inner 10–14 × 5–7
Ovary size, wings excluded (mm)	12–15 × 4–5	*Ca.* 8 × 3
Wings on ovary body	Lateral wings 5.5–8 mm wide, abaxial wing 10–13 mm wide	Lateral wings ca. 4 mm wide, abaxial wing ca. 7 mm wide
Tip of wings	With succulent bristles	Glabrous
**Distribution**	Northern Vietnam	Malay peninsula

**Figure 4:**
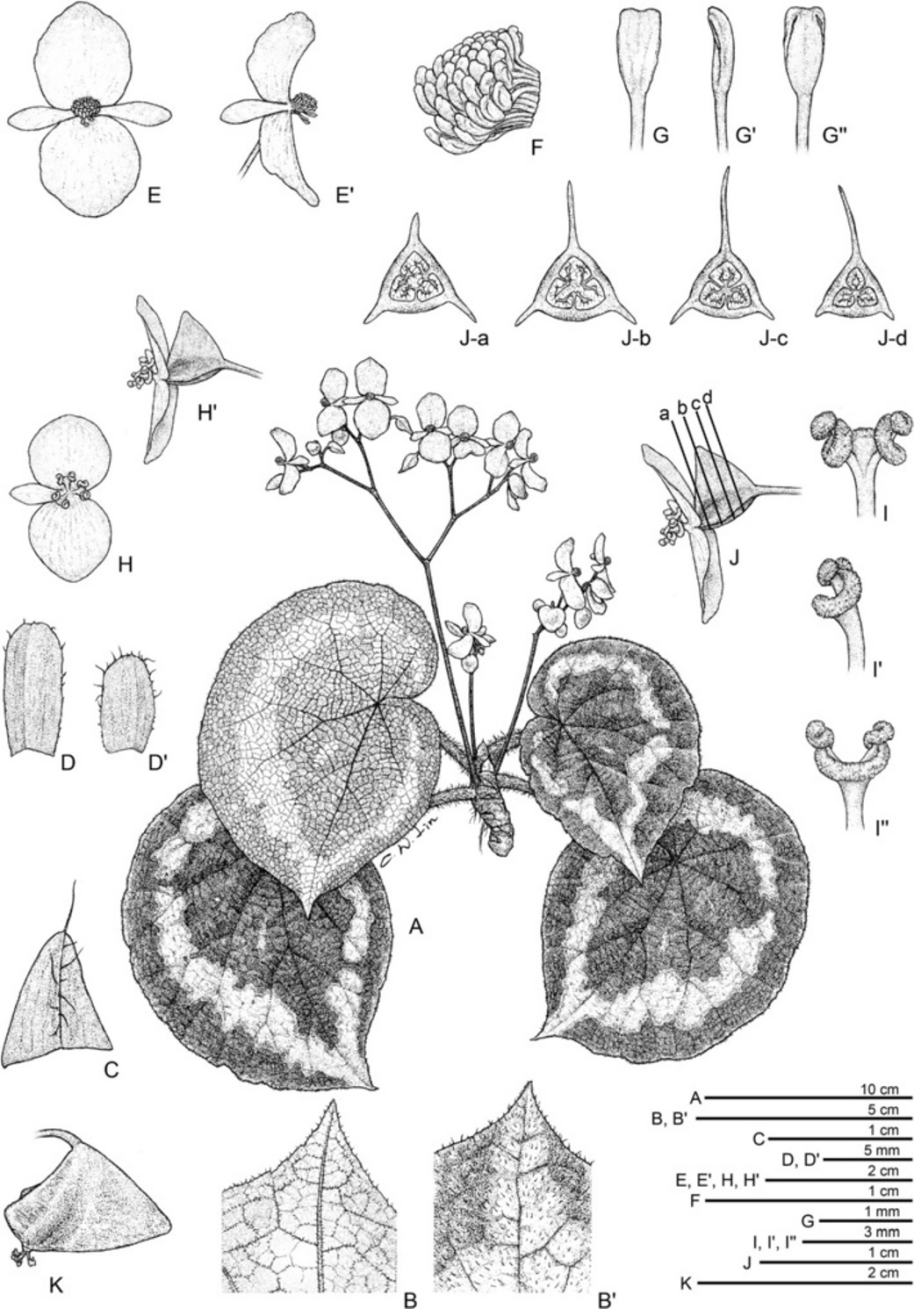
*Begonia circularis* C.-I Peng & C. W. Lin. **A**. Habit; **B**, **B**’. Portion of leaf, abaxial and adaxial surface; **C**. Stipule, abaxial view; **D**, **D**’. Bract and bracteole; **E**, **E**’. Staminate flowers; **F**. Androecium; **G**, **G**’, **G**”. Stamen; **H**, **H**’. Pistillate flowers; **I**, **I**’, **I**”. Style and stigmatic band; **J**. Serial cross sections of an immature capsule; **K**. Capsule. All from *Peng 22610* (HAST).

**Figure 5:**
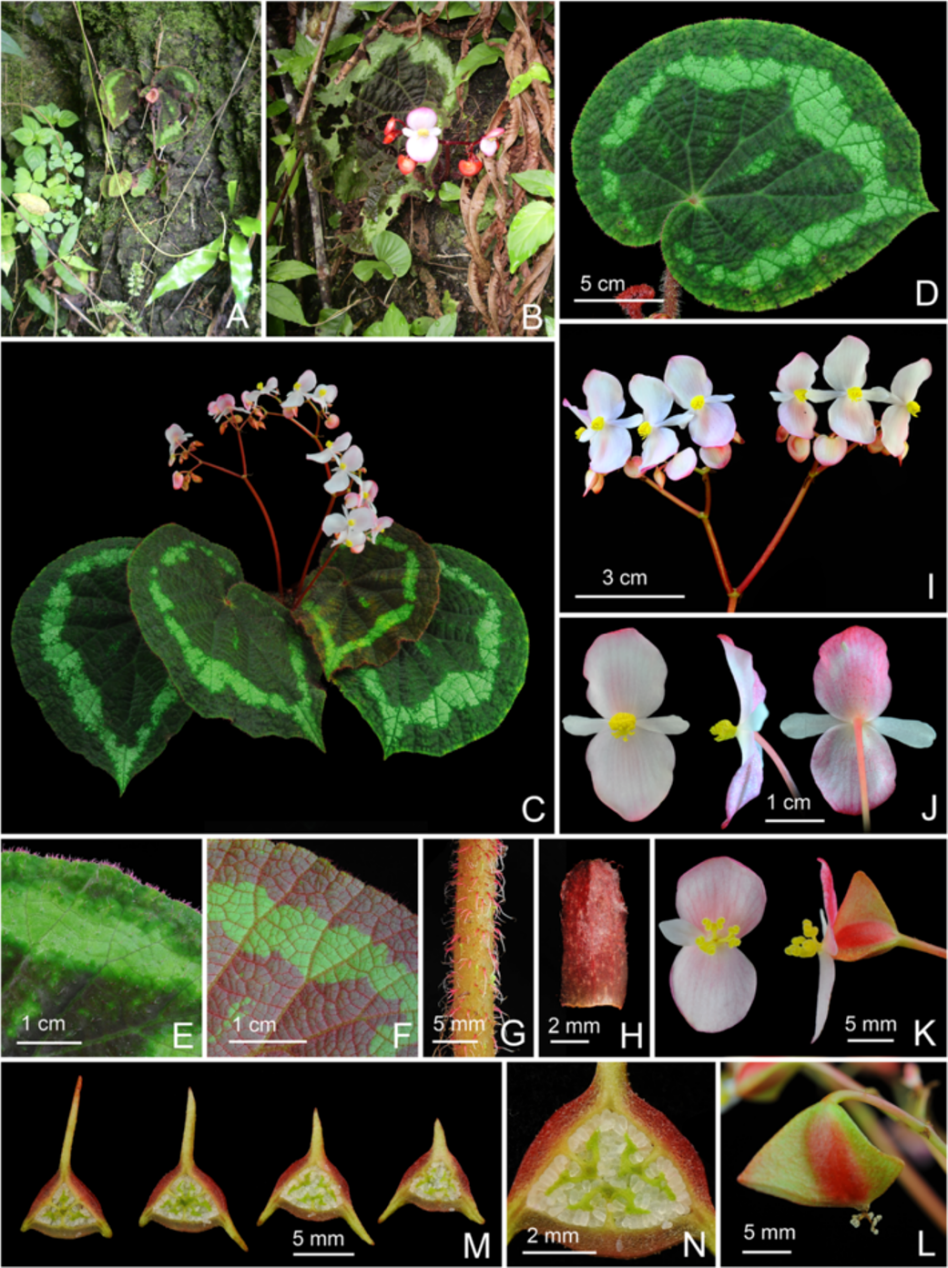
*Begonia circularis* C.-I Peng & C. W. Lin*.* **A**, **B**. Habitat; **C**. Habit; **D**, Leaf; **E**, **F**. Portion of leaf, adaxial and abaxial surface; **G**. Petiole; **H**. Bract; **I**. Inflorescence; **J**. Staminate flowers; **K**. Pistillate flowers; **L**. Capsule; **M**. Serial cross section of immature capsule, **N**. Same, closer view. All from *Peng 22610* (HAST).

**2.** ***Begonia circularis*** C.-I Peng & C. W. Lin, sp. nov. (sect. *Coelocentrum*) **—TYPE:** VIETNAM. Cao Bang Province, Thach An District, N 22°30’49”, E 106°22’40”. Seeds collected and presented by Mr. Etsuo Kobayashi. Type collection made from plants raised from seeds, 7 July 2014, *Peng 22610* (holotype: HAST) (Figures 4 and 5).

Herb, monoecious, rhizomatous. **Rhizome** stout, creeping, 1.2–2 cm thick, internodes congested, villous near the petiole insertion. **Stipules** deciduous, reddish, ovate-triangular, ca.1 cm long, 0.8 cm wide, strongly keeled, abaxially villous along midrib, apex aristate, arista ca. 0.5 cm long. **Petioles** red to reddish-green, terete, 5–9.5 (−14) cm long, 0.6–0.8 cm thick, densely pinkish villous. **Leaves** alternate; blade asymmetric, widely ovate to widely elliptic, 13.5–21 cm long, 9.5–17 cm wide, broad side 6–10 cm wide, basal lobes cordate, 4.8–7.5 cm, apex caudate, base strongly oblique-cordate, margin repand to denticulate, thickly chartaceous, densely magenta hispid, surface slightly rugose between veins, adaxially olive-green to dark green, embellished with a wide silvery green to lemon green ring and adorned with small similar-colored patches between primary palmate veins; abaxially reddish to pale magenta, with a ring of pale green corresponding to the pattern on adaxial surface; venation palmate with 6–8 primary veins, midrib distinct, with 2 or 3 secondary veins on each side, secondary veins branching dichotomously or nearly so, tertiary veins weakly percurrent or reticulate; all venation prominently raised and densely magenta hispid abaxially. **Inflorescences** axillary, dichasial cymes branched 3–5 times; peduncle 9.5–16 cm long, subglabrous; bracts and bracteoles caducous, reddish, oblong to ovate, ca. 0.8 cm long, 0.25–0.4 cm wide, boat-shaped, margin sparsely shortly fimbriate, bracteoles similar but smaller. **Staminate flower:** pedicel 0.9–2 cm long, tepals 4, outer 2 broadly ovate, 14–19 mm long, 13–16 mm wide, abaxially reddish, sparsely red setulose to glabrous, inner 2 oblanceolate, pinkish to white, 10–13 mm long, 2–4.5 mm wide; androecium zygomorphic, golfclub-shaped, ca. 5 mm across; stamens 45–65; anthers yellow, slightly compressed, obovate, ca. 1.3 mm long, apex retuse, filaments longer than anthers, slightly fused at base. **Pistillate flower:** pedicel 0.7–1 cm long, tepals 3, pinkish-white, outer 2 widely obovate to orbicular, 7–17 mm long, 8–13 mm wide, inner 1 narrowly elliptic, 5–12 mm long, 3–4 mm wide; ovary reddish, trigonous-ellipsoid, 6–10 mm long, 3–4 mm thick (wings excluded), with sparse sessile or subsessile glands, 3-winged, wings unequal, reddish-yellow, shallowly triangular, sparse subsessile-glandular, lateral wings 2–4 mm wide; abaxial wing crescent-shaped, entire, 6–10 mm wide; styles 3, fused at base, yellow, ca. 4 mm long, stigma spirally twisted. **Capsule** trigonous-ellipsoid, 10–17 mm long, 4–9 mm thick (wings excluded), reddish when fresh; wings unequal, shallowly triangular, lateral wings ca. 5 mm wide, abaxial wing 7–17 mm wide. Somatic chromosome number, 2*n* = 30.

#### Distribution and ecology

Endemic to Thach An District, Cao Bang Province, Vietnam (Figure 3), near the border between Vietnam and China. Growing in cracks of mossy rocks on semi-shaded limestone cliffs in evergreen broad-leaved forest, elevation ca. 600 m.

#### Etymology

The specific epithet refers to the circular foliar variegation of the new species.

#### Notes

*Begonia circularis* somewhat resembles *B. lanternaria* Irmsch. and *B. picturata* Yan Liu, S. M. Ku & C.-I Peng (SW Guangxi, China), all members of sect. *Coelocentrum*, in the variegated foliage. A comparison of salient features of the three species is shown in Table 2.

**Table 2:** **Camparison of** ***Begonia circularis*** **with** ***B. lanternaria*** **and** ***B. picturata***

	***B. circularis***	***B. lanternaria***	***B. picturata***(Liu et al.[2005:]figures one and two)
**Leaf**			
Maculation	A pale ring ca. 1.5–2 cm from the margin against a dark green background	With digitate, green or dark green bands along major veins and leaf margin	With digitate, dark brown bands along major veins and leaf margin
Adaxial surface	Hispid	Puberulous	Villous-setose or tomentose-setose, bullate at base
Abaxial surface	Hispid on all veins	Villous on major veins	Densely shortly villous, particularly on veins
**Bract**			
Shape	Oblong to ovate, boat-shaped, margin sparsely shortly fimbriate	Elliptic to broadly-ovate, margin glandulose-ciliate	Ovate, oblong to rounded, margin ciliate
Length (mm)	6–10 × 2.5–4	5–9 × 4–6.5	4.5–15 × 4.5–8
**Male flower**			
Outer tepal size (mm)	14–19 × 13–16	7–9 × 6–8	14–22 × 14–17
Inner tepal size (mm)	10–13 × 2–4	5.5–6 × 2–3	10–19 × 4.–5.5
Vestiture on outer tepals	Sparsely setulose to glabrous	Glandulose-pilose	Villous-setose
**Female flower**			
Ovary	With minute sessile glands	Glandulose-pilose	Villous-setose or hispid-setose
Wings on ovary	Deltate	Crescent-shaped	Crescent-shaped

**Figure 6:**
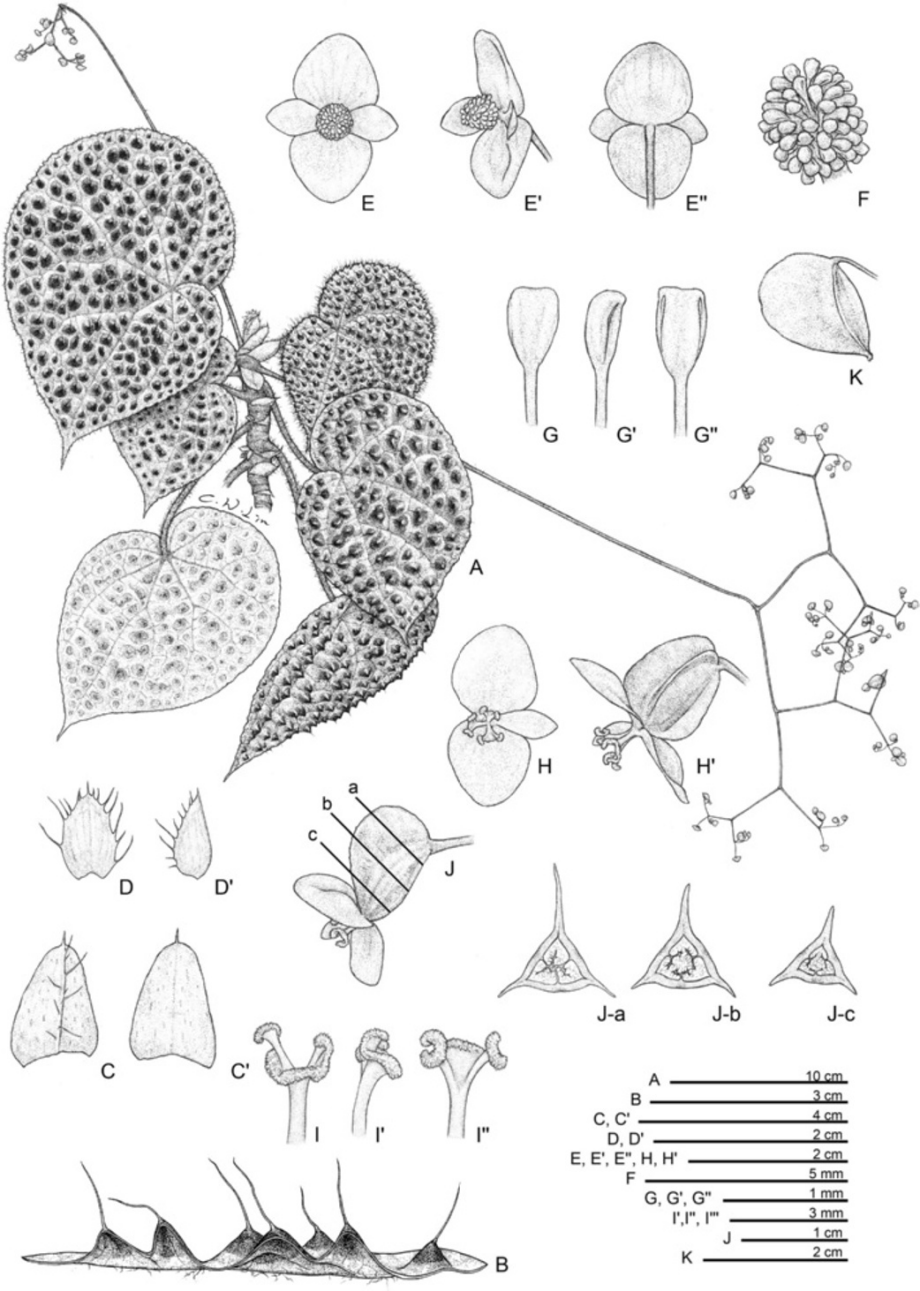
*Begonia melanobullata* C.-I Peng & C. W. Lin. **A**. Habit; **B**. Cross section of leaf, showing bullae; **C**, **C**’. Stipule, both surface views; **D**, **D**’. Bract, abaxial and side views; **E**, **E**’, **E**”. Staminate flowers; **F**. Androecium; **G**, **G**’, **G**”. Stamen; **H**, **H**’. Pistillate flowers; **I**, **I**’, **I**”. Style and stigmatic band; **J**. Cross section of an immature capsule; **K**. Capsule. All from *Peng 22609* (HAST).

**Figure 7:**
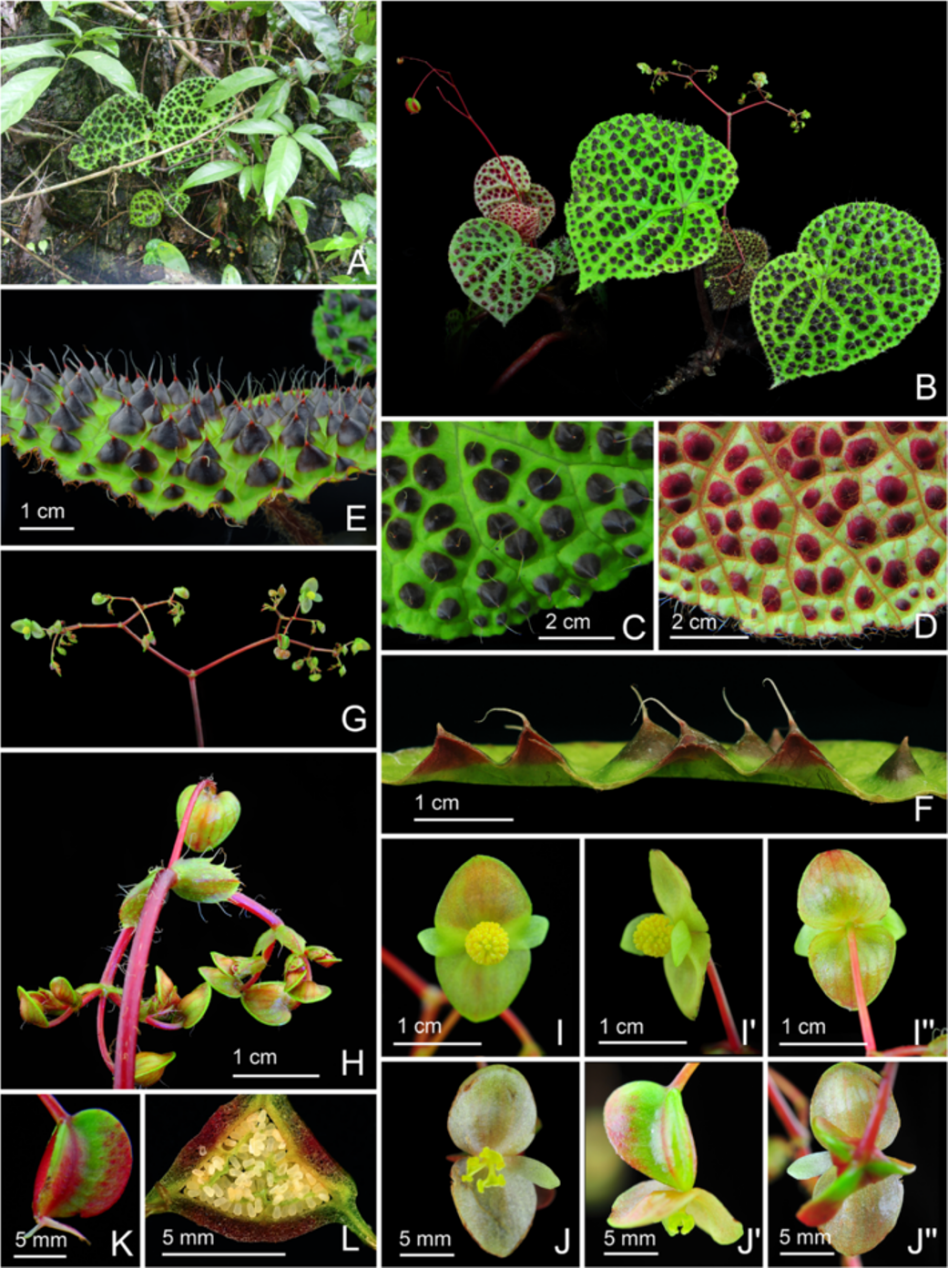
*Begonia melanobullata* C.-I Peng & C. W. Lin*.* **A**, Habitat; **B**. Habit; **C**, **D**. Portion of leaf, adaxial and abaxial surface, **E**. Same, leaf side view, showing conical bullae, each tipped by an erect hair; **F**. Leaf cross section; **G**. Inflorescence; **H**. Young inflorescence, showing bracts; **I**, **I**’, **I**”. Staminate flowers; **J**, **J**’, **J**”. Pistillate flowers; **K**. Capsule; **L**. Cross section of an immature capsule. All from *Peng 22609* (HAST).

**3.** ***Begonia melanobullata*** C.-I Peng & C. W. Lin, sp. nov. (sect. *Coelocentrum*) **—TYPE:** VIETNAM. Cao Bang Province, Thach An District, N 22°31’23”, E 106°20’41”. Living collection made on June 29, 2010; type specimens pressed from plants cultivated in the experimental greenhouse in Academia Sinica, Taiwan, 22 April 2014, *Ching-I Peng 22609* (holotype: HAST; isotypes: E, HN) (Figures 6 and 7).

Herbs, monoecious, perennial, rhizomatous. **Rhizome** stout, creeping, to 50 cm long,1–2 (−2.5) cm thick, internodes 1–2 cm long, villous near petiole insertion. **Stipules** yellowish to pale green, ovate-triangular, 1.4–2.5 cm long, 1–1.8 cm wide, strongly keeled, abaxially hairy along midrib, apex aristate, arista *ca.* 0.2 cm long. **Petiole** red to olive-green, terete, 8–21 cm long, 0.4–0.8 cm thick, densely white villous when young, turning brownish tomentose, sometimes subglabrous with age. **Leaves** alternate, blade asymmetric, widely ovate to widely elliptic, 13–21 cm long, 9–15 cm wide, broad side 6.5–12 cm wide, basal lobes cordate, 5–8.5 cm, apex caudate, base strongly oblique-cordate, margin repand, thick chartaceous, villous when juvenile, adaxially emerald green to yellowish green, surface densely bullate, bulla dark maroon, conical, 2–8 mm high, 3–8 mm across, tipped with a velutinous hair 6–10 mm long, abaxially pale green, reddish on veins and bullae, brownish tomentose on veins; venation palmate with 7–9 primary veins, midrib distinct, with 2–4 secondary veins on each side, tertiary veins reticulate or percurrent, minor veins reticulate. **Inflorescences** arising directly from rhizome, dichasial cymes branched 4–6 times; peduncle 15–38 cm long, tomentose; bracts at cyme base pale green, ovate, ca. 1 cm long, 0. 5 cm wide, boat-shaped, veins reddish, margin fimbriate, bracteoles on upper nodes similar but gradually smaller. **Staminate flower:** pedicel ca. 1.5 cm long, tepals 4, outer 2 broadly ovate, 6–9 mm long, 7–9 mm wide, abaxially greenish-reddish, sparsely setulose, adaxially yellowish-green, inner 2 greenish, elliptic, 5–7 mm long, 3–4 mm wide; androecium actinomorphic, spheroid, 2.5–3.5 mm across; stamens 65–80; anthers yellowish green to gold yellow, slightly compressed, obovate, 0.5–0.8 mm long, apex retuse to obtuse; filaments fused at base into a column ca. 1.5 mm long; **Pistillate flower:** pedicel ca. 1.5 cm long, tepals 3, outer 2 suborbicular or broadly ovate, abaxially reddish, adaxially yellowish-pinkish, 7–8 mm long, 7–8 mm wide, inner 1 yellowish-pinkish, elliptic, 4–6 mm long, 0.2–0.3 cm wide; ovary trigonous-ellipsoid, 8–10 mm long, 4 mm thick (wings excluded), reddish, sparsely dotted with sessile glands, 3-winged, wings unequal, reddish-green, lateral wings 2–3 mm wide, abaxial wing crescent-shaped, 8–10 mm long, 4–6 mm wide; styles 3, fused at base, yellow or greenish, ca. 3 mm long, stigma spirally twisted. **Capsule** trigonous-ellipsoid, 12–19 mm long, 5–8 mm thick (wings excluded), greenish or reddish when fresh; wings unequal, lateral wings 2–4 mm wide, abaxial wing crescent-shaped, 5–7 mm wide. Somatic chromosome number, 2*n* = 30.

#### Distribution and ecology

Endemic to Thach An District, Cao Bang Province, Vietnam (Figure 3), near the border between Vietnam and China. On semi-shaded wet cliffs or steep limestone slopes in evergreen broad-leaved forest, elevation at 300–400 m.

#### Etymology

The specific epithet refers to the color of the bullae on leaf surface.

#### Notes

*Begonia melanobullata* resembles *B. ferox* C.-I Peng & Yan Liu (Guangxi, China) and *B. nahangensis* Aver. & H. Q. Nguyen (Northern Vietnam) in having blackish bullae on leaf upper surface. However, *Begonia melanobullata* is sharply distinct in other aspects. The new species is similar to *B. ferox*, differing by the leaves widely ovate to widely elliptic (vs. ovate), peduncle 15–38 cm (vs. 5–13 cm) long, inflorescence branched 4–6 (vs. 3–4) times, staminate flower tepals greenish (vs. yellowish-reddish) and smaller anthers. *Begonia melanobullata* also resembles *B. nahangensis*, differing in the leaf apex acuminate (vs. obtuse), staminate flower greenish (vs. white to pinkish); tepals of pistillate flowers yellowish-pinkish (vs. light olive-green), suborbicular or broadly ovate (vs. broadly reniform). A comparison of salient features of the three species is shown in Table 3. *Begonia melanobullata* is known only from limestone areas in Cao Bang Province.

**Table 3:** **Camparison of** ***Begonia melanobullata*** **with** ***B. ferox*** **and** ***B. nahangensis***

	***B. melanobullata***	***B. ferox***(Peng et al.[2013]: figures five and six)	***B. nahangensis***(Averyanov and Nguyen[2012]: figure seven)
**Stipule**			
Length	1.4–2.5 cm	1–1.7 cm	0.4–0.6 cm
**Leaf**			
Apex	Acuminate	Acuminate	Obtuse to nearly rounded
Shape	Widely ovate to widely elliptic	Ovate	Broadly oblique-ovate or oblique-reniform
Conical bullae on upper surface	Present on all leaves; erect hairs tipping bullae persistent	Progressively developed on leaves as the plant matures; hairs on bullae soon deciduous	Unknown
**Inflorescence**			
Peduncle length (cm)	15–38	5–13	(8–) 10–12 (−14)
**Male flower**			
Tepal color	Greenish	Pale pinkish-yellow	White to light pink
Tepal size (mm)	Outer 6–9 × 7–9, inner 5–7 × 3–4	Outer 9–11 × 6–11, inner 7–11 × 4	Outer 8–9 in diam., inner 8–9 × 2.5–3.5
Another length (mm)	0.5–0.8	Ca. 1	Ca. 0.5
**Female flower**			
Tepal color	Yellowish-pinkish	Pinkish-white	Light olive-green
Tepal size (mm)	Outer 7–8 × 7–8, inner 4–6 × 2–3	Outer 8–11 × 7–11 mm, inner 8–9 × 3–4	Outer (4.5) 5–6 (7) × (8) 9–11 (12) mm, inner 5–6 × 3–3.5
**Capsule**			
Size	1.2–1.9 cm long; abaxial wing crescent-shaped, 0.5–0.7 cm wide	1–1.5 cm long; abaxial wing crescent-shaped, 0.6–0.9 cm wide	0.8–1 cm long; abaxial wing oblique-triangular, 0.4 cm wide
**Distribution**	Northern Vietnam	Guangxi, China	Northern Vietnam

**Figure 8:**
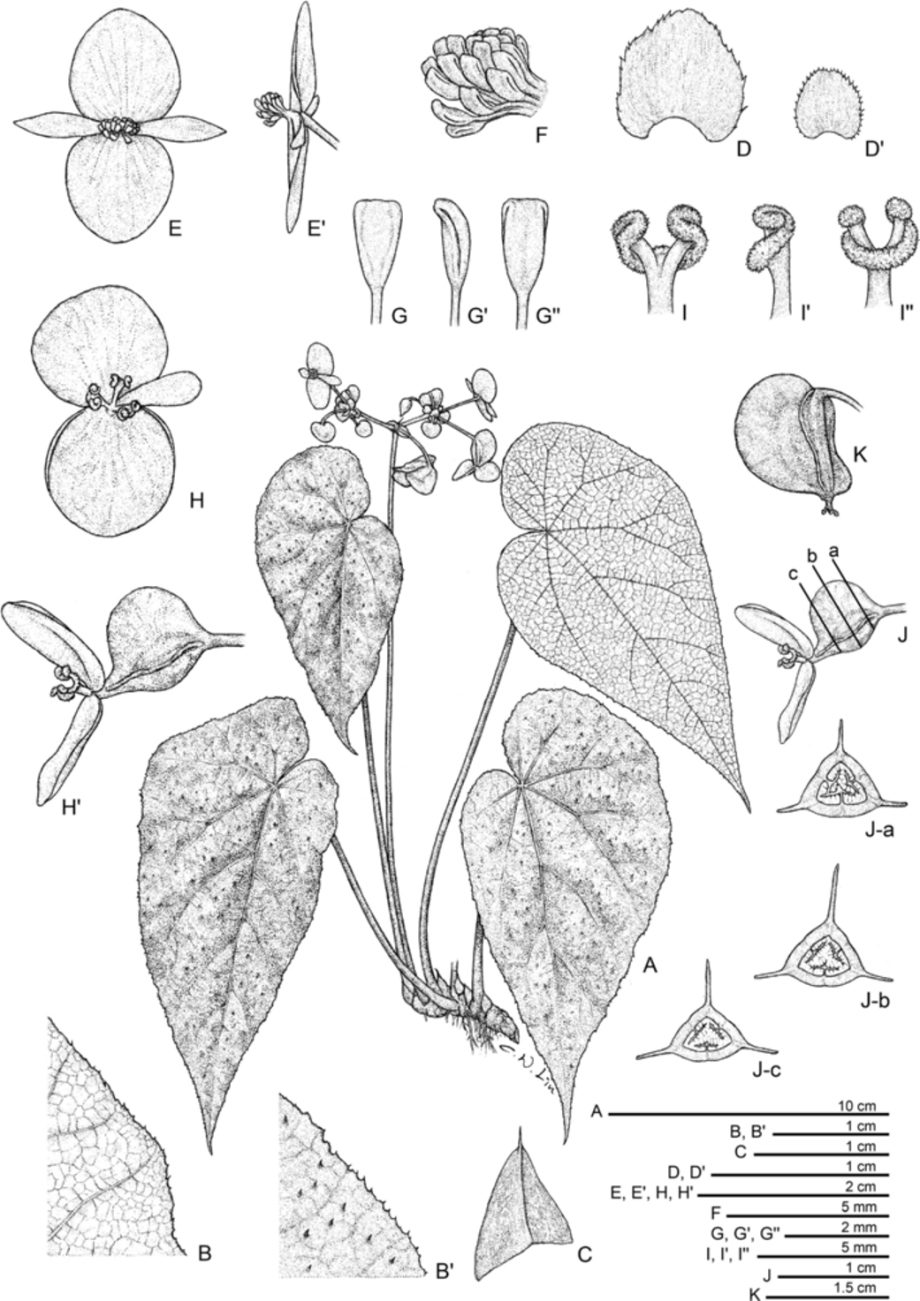
*Begonia langsonensis* C.-I Peng & C. W. Lin. **A**. Habit; **B**, **B**’. Portion of leaf, abaxial and adaxial surface; **C**. Stipule, abaxial view; **D**, **D**’. Bract and bracteole; **E**, **E**’. Staminate flowers; **F**. Androecium; **G**, **G**’, **G**”. Stamen; **H**, **H**’. Pistillate flowers; **I**, **I**’, **I**”. Style and stigmatic band; **J**. Serial cross section of immature capsule; **K**. Capsule. All from *Peng 21946* (HAST).

**Figure 9:**
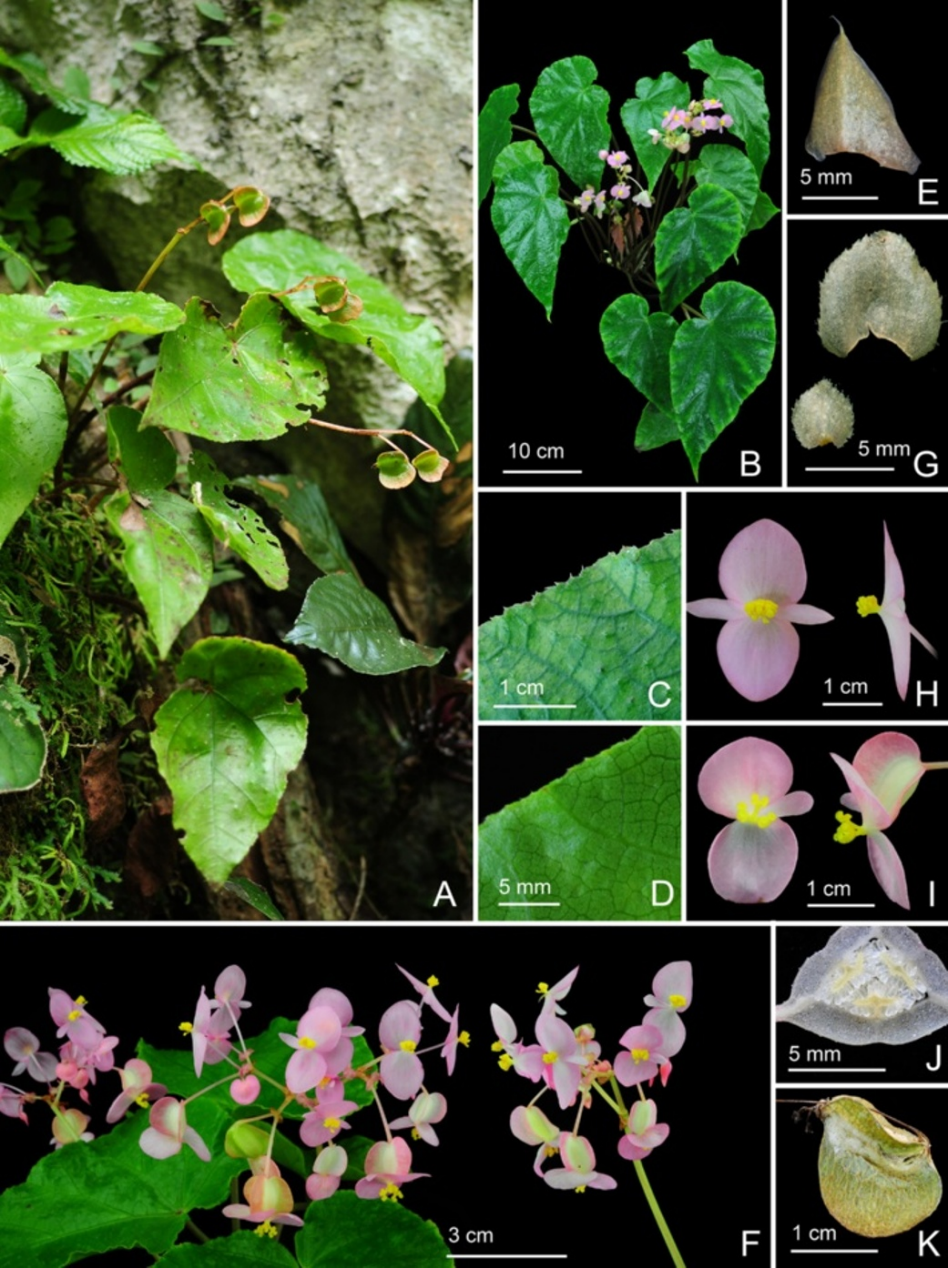
*Begonia langsonensis* C.-I Peng & C. W. Lin. **A**. Habitat; **B**. Habit; **C**, **D**. Portion of leaf, adaxial and abaxial surface; **E**. Stipule; **F**. Inflorescence; **G**. bract and bracteole; **H**. Staminate flowers; **I**. Pistillate flowers; **J**. Cross section of immature capsule; **K**. Capsule. All from *Peng 21946* (HAST).

**4.** ***Begonia langsonensis*** C.-I Peng & C. W. Lin, sp. nov. (sect. *Coelocentrum*) **—TYPE:** VIETNAM. Lang Son Province, Huu Lung District, Huu Lien Commune, Tam Lai Village. N 21°39’60”, E 106°22’53”, elev. ca. 140 m. Living collection made on 20 November 2008; type specimens pressed from plants cultivated in the experimental greenhouse in Academia Sinica, Taiwan, 15 November 2012, *Peng 21946-A* (holotype: HAST; isotypes: A, E, HN, MO) (Figures 8 and 9).

Herb, monoecious, rhizomatous. **Rhizome** creeping, to 10 cm or longer, 0.6–1.7 cm thick, internodes congested, glabrous. **Stipules** deciduous, reddish pale green, widely ovate-triangular, 0.7–1 cm long, 0.7–1.1 cm wide, herbaceous, strongly keeled, glabrous, apex aristate, arista ca. 0.3 cm long. **Petioles** brownish green, terete, 13–25 cm long, 0.3–0.7 cm thick, glabrous. **Leaves** alternate, blade asymmetric, obliquely ovate, 11–22.5 cm long, 6–13.5 cm wide, broad side 3.3–6.8 cm wide, basal lobes cordate, 2.7–5.5 cm long, apex attenuate, caudate, base strongly oblique-cordate, margin serrulate and puberulous, thickly chartaceous, adaxially uniformly emerald green, very sparsely minutely scabrescent, abaxially pale green, sparingly puberulous or subglabrous; venation palmate with 6–8 primary veins, midrib distinct, secondary veins 2–4 on each side, minor veins reticulate, all venation prominently raised on upper surface, major and secondary veins prominent on lower surface. **Inflorescences** axillary, arising directly from rhizome, dichasial cymes branching 4–6 times; peduncle 12–26 cm long, subequal to leaves, glabrous; bracts and bracteoles caducous, white, pale green to pinkish, bracts widely ovate to very widely ovate, 0.6–0.9 cm long, 0.6–0.8 cm wide, flat, margin denticulate to biserrate, puberulous or glandular hairy, bracteoles widely ovate to orbicular, margin denticulate, glandular hairy, gradually smaller toward upper part of the inflorescence. **Staminate flower:** pedicel ca. 2.2 cm long, tepals 4, outer 2 broadly ovate to orbicular, 10–15 mm long, 11–14 mm wide, abaxially pink, glabrous, adaxially pinkish, inner 2 pinkish, lanceolate, 6–9 mm long, 2.5–3.5 mm wide; androecium zygomorphic, ca. 5 mm across; stamens 25–40; filaments coalescent, not forming a stalk at base; anthers yellow, slightly compressed, obovate, ca. 1.5 mm long, apex retuse, shorter than filaments. **Pistillate flower:** pedicel 1.8–2.6 cm long, tepals 3, outer 2 pink or pinkish on both surfaces, orbicular, 11–15 mm long, 13–18 mm wide, inner 1 pinkish, oblanceolate, 7–11 mm long, 3–4 mm wide; ovary whitish green to pinkish, trigonous-ellipsoid, 12–15 mm long, ca. 5 mm thick (wings excluded), with sparse sessile glands, 3-winged, wings unequal, pinkish, lateral wings narrower, 6–4.5 mm wide, abaxial wing crescent-shaped, 5–7 mm wide; styles 3, fused at base, yellow, 4–5.5 mm long, stigma spirally twisted. **Capsule** trigonous-ellipsoid, 13–16 mm long, ca. 5 mm thick (wings excluded), greenish or reddish when fresh; wings unequal, lateral wings 3–5 mm wide, abaxial wing crescent-shaped, 7–9 mm wide. Somatic chromosome number, 2*n* = 30.

#### Distribution and ecology

Endemic to Huu Lung Protected Area, Huu Lien District, Lang Son Province, Vietnam (Figure 3). On semishady cliff face or steep limestone slopes in evergreen broad-leaved forest, elevation ca. 125–165 m.

#### Etymology

The specific epithet refers to Lang Son Province, where the new species was discovered.

#### Notes

*Begonia langsonensis* somewhat resembles *B. ornithophylla* Irmsch. (southwest Guangxi, China) in leaf shape and aspect. However, *B. langsonensis* is nearly glabrous throughout, whereas *B. ornithophylla* has sericeous petiole, puberulous abaxial blade, as well as velutinous to villous pedicel and peduncle. In addition, *B. langsonensis* differs from the latter in the bracts widely to very widely ovate (vs. ovate-triangular) as well as the primary and secondary veins on leaf adaxial surface elevated (vs. sunken).

**Figure 10:**
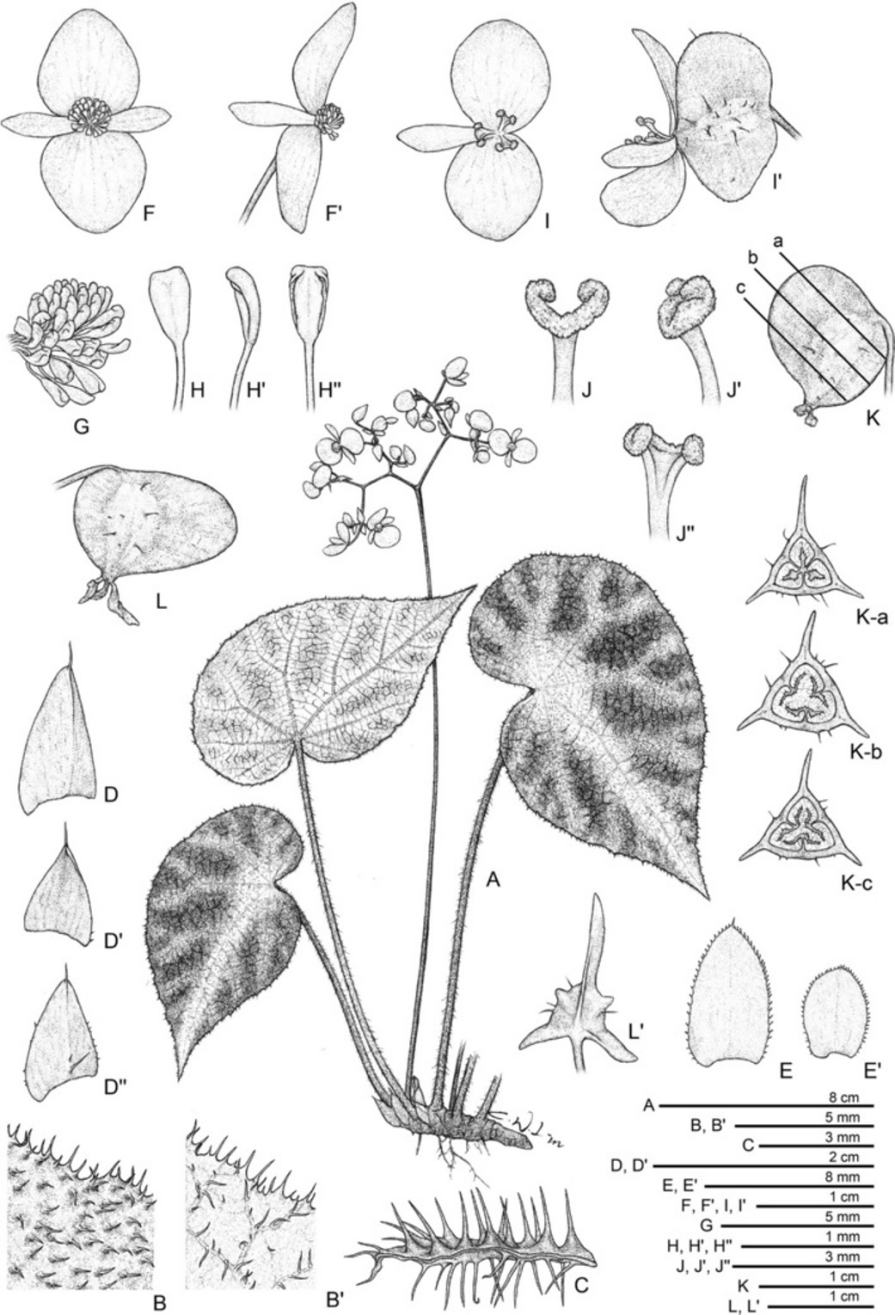
*Begonia locii* C.-I Peng, C. W. Lin & H. Q. Nguyen. **A**. Habit; **B**, **B**’. Portion of leaf, adaxial and abaxial surface; **C**. Cross section of leaf; **D**, **D**”. Stipule, abaxial view, **D**’. adaxial view; E. Bract and bracteole; **F**, **F**’. Staminate flowers; G. Androecium; **H**, **H**’, **H**”. Stamen; **I**, **I**’. Pistillate flowers; **J**, **J**’, **J**”. Style and stigmatic band; **K**. Serial cross section of an immature capsule; **L**. Capsule, **L**’. Capsule, back view, showing the occasional presence of 2 additional, reduced wings. All from *Peng 21943* (HAST).

**Figure 11:**
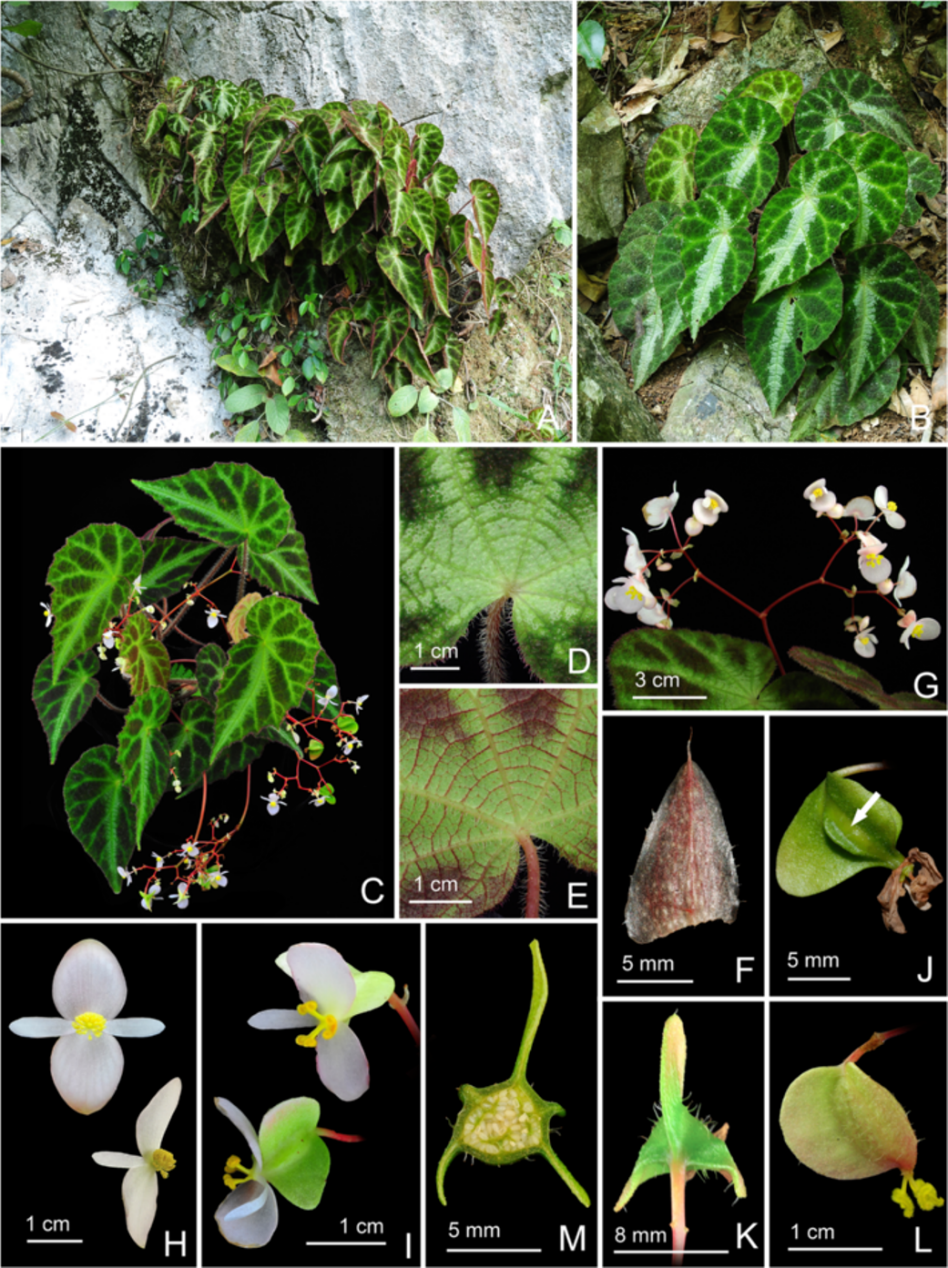
*Begonia locii* C.-I Peng, C. W. Lin & H. Q. Nguyen*.* **A**, **B**. Habit and habitats; **C**. Habit; **D**, **E**. Portion of leaf, adaxial and abaxial surface; **F**. Stipule; **G**. Inflorescence; **H**. Staminate flowers; **I**. Pistillate flowers; **J**. Capsule, side view, showing the additional reduced wing (arrows); **K**. Capsule, back view, showing 3 wings; **L**. Capsule; **M**. Cross section of an immature capsule. All from *Peng* *21943* (HAST).

**5.** ***Begonia locii*** C.-I Peng, C. W. Lin & H. Q. Nguyen, sp. nov. (sect. *Coelocentrum*) **—TYPE:** VIETNAM. Lang Son Province, Huu Lung District, Huu Lien Commune, Tam Lai Village, N 21°39’60”, E 106°22’53’, elev. ca. 185 m. Living collection made on 20 November 2008; type specimens pressed from plants cultivated in the experimental greenhouse in Academia Sinica, Taiwan, 8 April 2010, *Peng 21943-A* (holotype, HAST; isotype, A, E, HN, KEP, MO) (Figures 10 and 11).

Herb, monoecious, rhizomatous. **Rhizome** stout, creeping, to 30 cm long, 0.8 − 1.6 (−2) cm thick, internodes 0.5–1.3 cm long, subglabrous. **Stipules** deciduous, reddish to olive with red veins, ovate-triangular, 0.6–1.4 cm long, 0.5–0.85 cm wide, strongly keeled, abaxially sparsely velutinous along midrib, margin entire or sparsely ciliate-dentate, apex aristate, arista ca. 0.25 cm long. **Petioles** pale brownish-green to rwddish, terete, (11−) 20–25 (−33) cm long, 0.3–0.6 cm thick, densely villous. **Leaves** alternate, blade asymmetric, obliquely ovate, (9−) 11–22 cm long, 6.5–12.5 cm wide, broad side 3.2–7.4 cm wide, basal lobes cordate, 3.3–6.6 cm long, chartaceous, apex acuminate, base strongly obliquely cordate, margin denticulate and densely puberulous with pale magenta hairs, adaxially deep olive-green to maroon with light green zones along primary and secondary veins, sometimes embellished with small bits of silvery white spots, midrib veins forming a wide silvery white zone; upper surface densely covered by small raised cones each topped by a magenta hair, giving a wrinkled texture; abaxially pale green, purplish red between primary and secondary veins, reddish-white tomentose on all veins; venation palmate with 7–8 primary veins, midrib distinct, with 3–5 secondary veins on each side, tertiary veins red, percurrent or reticulate. **Inflorescences** axillary, arising directly from rhizome, dichasial cymes branched 3–4 times; peduncle 13–35 cm long, tomentose or subglabrous; bracts and bracteoles caducous, pale yellow-green, ovate to elliptic, orbicular, bracts ca. 0.8 cm long, 0.5 cm wide, margin gland-tipped denticulate, bracteoles similar but smaller. **Staminate flower:** pedicel ca. 1 cm long, tepals 4, outer 2 broadly ovate to orbicular, 9–12 mm long, 8–11 mm wide, abaxially white to pinkish, adaxially whitish, glabrous, inner 2 elliptic, white, 7–11 mm long, 3–4.5 mm wide; androecium zygomorphic, subglobose, ca. 4 mm across; stamens 35–60; filaments fused at base; anthers yellow, slightly compressed, obovate, ca. 1 mm long, apex retuse, shorter than filaments. **Pistillate flower:** pedicel ca. 1 cm long, tepals 3, outer 2 pinkish to white, suborbicular or broadly ovate, 8–11 mm long, 8–12 mm wide, inner 1 white, oblanceolate to obovate, 6–10 mm long, 3–5 mm wide; ovary trigonous-ellipsoid, sometimes strongly keeled at middle, 7–12 mm long, 3–4.5 mm thick (wings excluded), whitish, sparsely hirsute and glandular, 3-winged, wings whitish green to pinkish, unequal, lateral wings narrower, narrowly crescent-shaped to trapezium, 2.5–6 mm wide, abaxial wing crescent-shaped, 4.5–7 mm wide, sometimes with 2 very reduced wings running parallel along the side of the ovary, sparsely hirsute to glabrous, margin entire to hirsute; styles 3, fused at base, yellow to greenish, 3–4.5 mm long, stigma spirally twisted. **Capsule** trigonous-ellipsoid, 10–15 mm long, 4–5 mm thick (wings excluded), greenish or reddish when fresh, glabrous to hirsute; wings unequal, lateral wings 4–6 mm wide, abaxial wing crescent-shaped, 6–7 mm wide. Somatic chromosome number, 2*n* = 30.

#### Additional specimens examined

Vietnam. Lang Son, Huu Lung District, Huu Lien Commune, Tam Lai Village, E106°22’53”, N21°39’60”, elevation ca. 185 m, *Peng 21938* (HAST).

#### Distribution and ecology

*Begonia locii* is endemic to Huu Lung Protected Area, Huu Lien District, Lang Son Province, Vietnam (Figure 3), occurring on semi-shaded limestone rock face, elevation 125–165 m.

#### Etymology

*Begonia locii* is named in honor of Professor Phan Ke Loc for his contribution to Vietnamese botany.

#### Notes

*Begonia locii* resembles *B. luochengensis* S.M. Ku, C.-I Peng & Yan Liu from Guangxi, China in leaf shape and maculation pattern, but is clearly distinct from the latter in the percurrent and spiderweb-like leaf venation (vs. pinnate) near the attachment of the petioles; upper leaf surface densely covered with small raised cones (vs. nearly flat); and hirsute (vs. glabrous) ovaries. The new species is also similar to *B. pengii* S. M. Ku & Yan Liu in leaf maculation pattern, vestiture, and hirsute ovaries, differing by the leaf basifixed (vs. peltate); bract margin denticulate and glandular (vs. denticulate and ciliate). A comparison of salient characters of the three species, all of sect. *Coelocentrum*, is shown in Table 4.

**Table 4:** **Camparison of** ***Begonia locii*** **with** ***B. luochengensis*** **and** ***B. pengii***

	***B. locii***	***B. luochengensis***(Ku et al.[2004]: figures four and five)	***B. pengii***(Ku et al.[2008]: figures one and two)
**Leaf**			
Attachment	Basifixed	Basifixed	Peltate
Venation near petiole	Spider-web like	Pinnate	Spider-web like
Adaxial vestiture	Scabrous, bullate at base	Villous	Scabrous, bullate at base
**Stipule**	Triangular-ovate, abaxially sparsely velutinous along midrib, margin entire or sparsely ciliate-dentate	Widely triangular-ovate, abaxially sparsely villous to glabrous, margin ciliate-dentate or long-ciliate	Narrowly triangular-ovate, glabrous, margin eciliate
**Bract**			
Margin	Gland-tipped denticulate	Sparsely ciliate-serrate.	Denticulate and ciliate
**Inflorescence**			
Peduncle	Tomentose to glabrous	Glabrous	Pilose or hispid-villous
**Female flower**			
Ovary	Hirsute	Glabrous	Pilose or villous-pilose
**Capsule**			
Body size (mm)	10–15 × 4–5	11–17 × 6–7	18–25 × 6–13
Width of lateral wings (mm)	4–6	3–4	2–3
Width of abaxial wing (mm)	6–7	10–12.5	7–11

**Figure 12:**
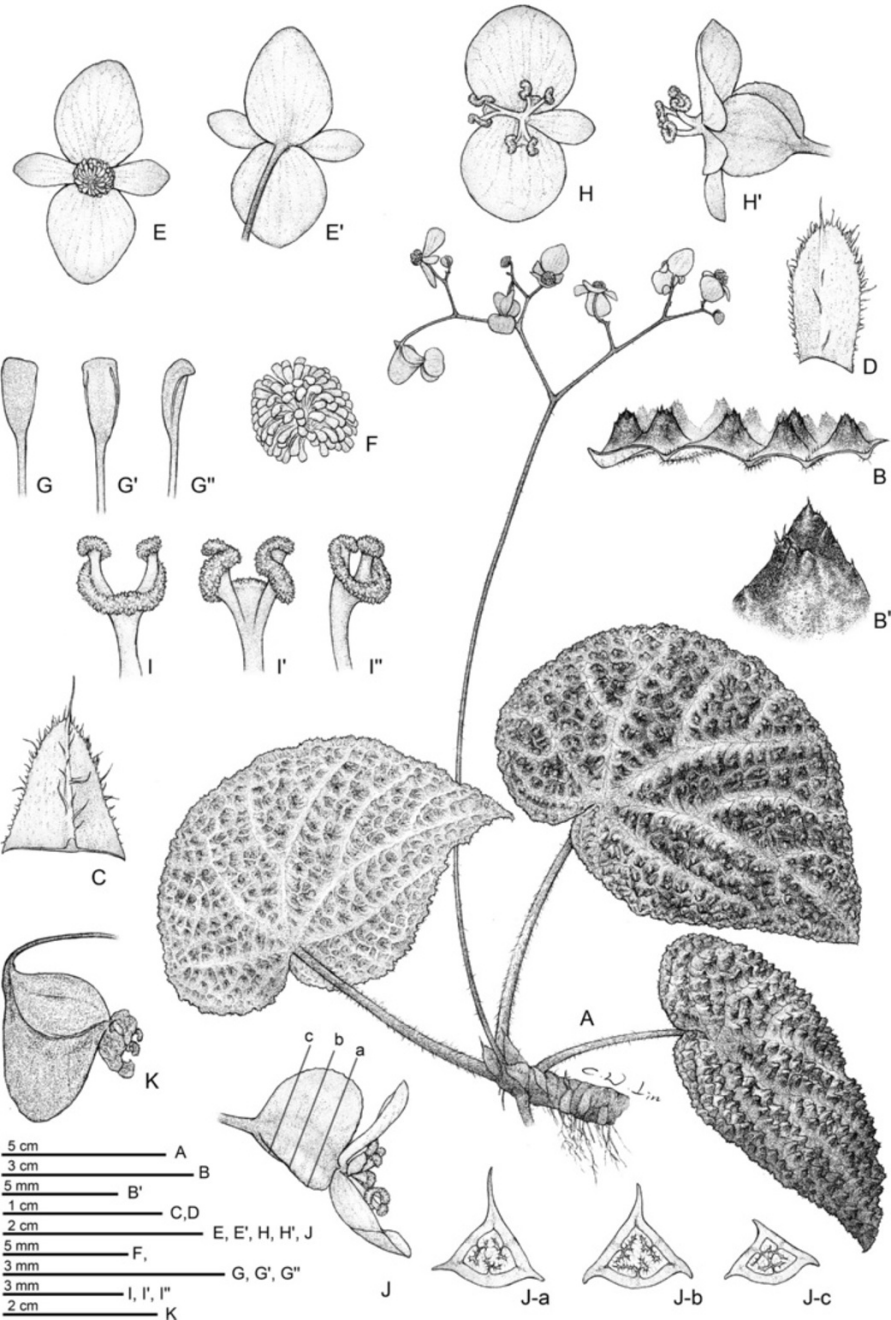
*Begonia montaniformis* C.-I Peng, C. W. Lin & H. Q. Nguyen*.* **A**. Habit; **B**. Cross section of leaf, **B**’. 2–4 tipped conic bulla; **C**. Stipule, abaxial view; **D**. Bract; **E**, **E**’. Staminate flowers; **F**. Androecium; **G**, **G**’, **G**”. Stamen; **H**, **H**’. Pistillate flowers; **I**, **I**’, **I**”. Style and stigmatic band; **J**. Cross section of an immature capsule; **K**. Capsule. All from *Peng 24609* (HAST).

**Figure 13:**
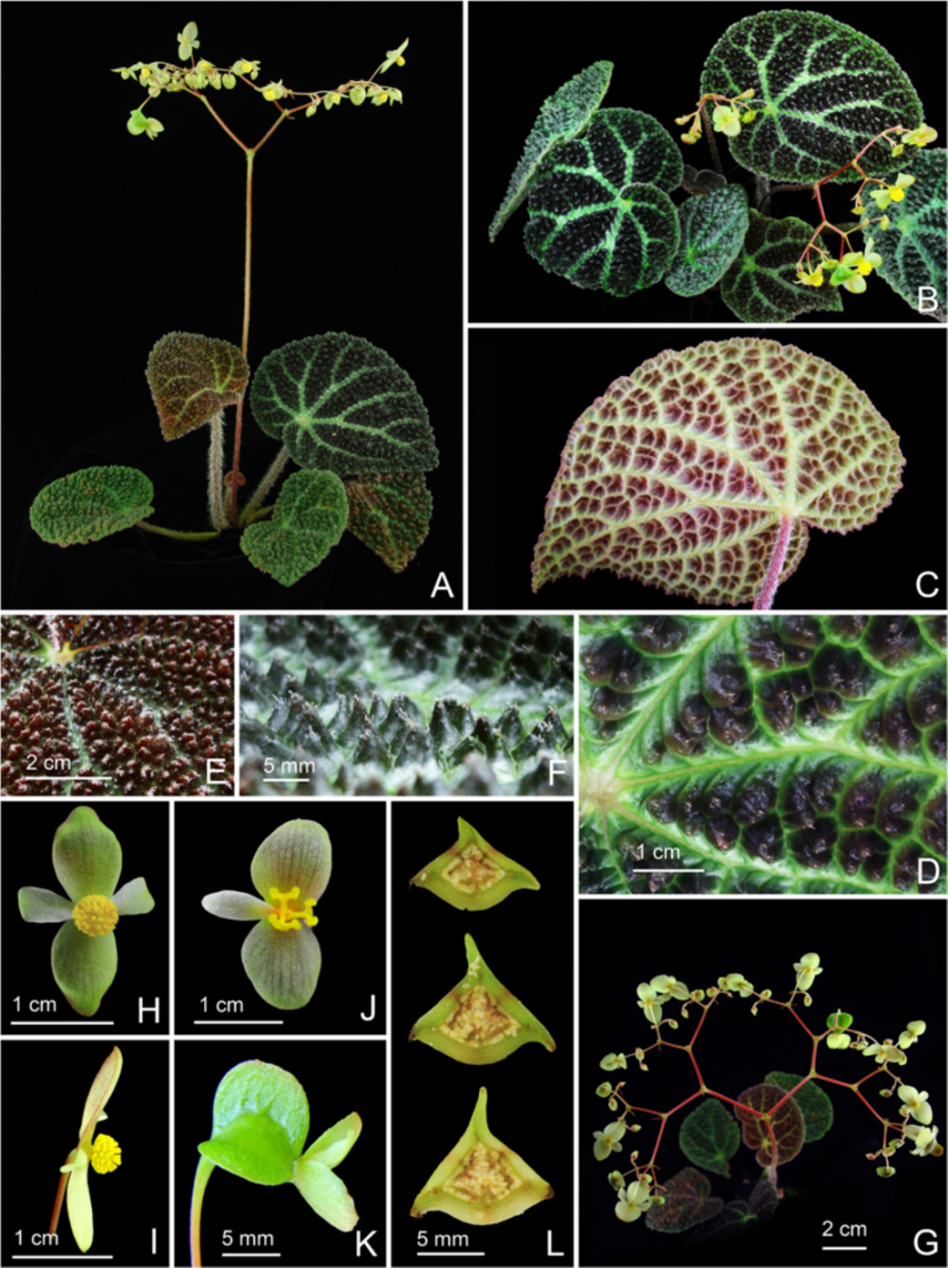
*Begonia montaniformis* C.-I Peng, C. W. Lin & H. Q. Nguyen*.* **A**, **B**. Habit; **C**. Leaf, abaxial surface; **D**, **E**, **F**. Portion of leaf, showing conic bullae on adaxial surface; **G**. Inflorescence; **H**, **I**. Staminate flowers; **J**, **K**. Pistillate flowers; **L**. Serial cross sections of an immature capsule. All from *Peng 24609* (HAST).

**6.** ***Begonia montaniformis*** C.-I Peng, C. W. Lin & H. Q. Nguyen, sp. nov. (sect. *Coelocentrum*) **—TYPE:** North VIETNAM. Thanh Hoa Prov., Ba Thuoc Distr., Co Lung Municipality, Eo Dieu village. Primary evergreen seasonal broad-leaved closed lowland forest on crystalline marble-like highly eroded limestone, very steep rocky slopes and cliffs of S exposition at elev. 450–600 m. Lithophytic herb on shady cliff. Introduced from home garden plants in Hanoi for cultivation in Academia Sinica. Type specimen pressed from the greenhouse plant on 5 June 2006. *Ching-I Peng 24609* (holotype: HAST; isotype: HN) (Figures 12 and 13).

Herbs, monoecious, perennial, rhizomatous. **Rhizome** stout, creeping, to 30 cm long, 1–2.3 cm thick, internodes 0.5–1.7 cm long, villous near the petiole insertion. **Stipules** pale green to pinkish, ovate-triangular, 0.7–1.4 cm long, 0.6–1.3 cm wide, strongly keeled, abaxially hairy along midrib, margin velutinous, apex aristate, arista ca. 0.2 cm long. **Petiole** red to greenish-purple, terete, 7–20 cm long, 0.4–0.7 cm thick, densely white villous. **Leaves** alternate, blade asymmetric, ovate to widely ovate, 11.5–19 cm long, 8–13.5 cm wide, broad side 6–8 cm wide, apex acuminate to acute, base strongly oblique-cordate, margin repand to shallowly denticulate and ciliate, thickly chartaceous to succulent, adaxially blackish-malachite green, purplish-olive or dark bluish-brown, with silvery green zone along primary and secondary veins, surface bullate, bulla conical, 0.4–0.8 (−1) cm high, 0.5–0.8 cm across, tipped by 2–4 (−6) peak-like hispidulous protrusions, abaxially pale green, reddish on hollow bullate region, densely brownish-white tomentose on veins; venation palmate with 7–9 primary veins, midrib distinct, 2–4 secondary veins on each side, tertiary veins percurrent or reticulate, sometimes spiderweb-like near petiole attachment, minor veins reticulate. **Inflorescences** axillary, arising directly from rhizome, dichasial cymes branching 3–8 times; peduncle 11–28 cm long, tomentose to subglabrous; bracts and bracteoles caducous, yellowish-green to reddish, bracts ovate, ca. 1 cm long, 0. 5 cm wide, boat-shaped, abaxially sparsely velutinous along midrib, margin densely tomentose, bracteoles similar to bracts but gradually smaller on upper part of the inflorescence. **Staminate flower:** pedicel 1.1–2 cm long, tepals 4, outer 2 broadly ovate to obovate-orbicular, 7–12 mm long, 6–10 mm wide, abaxially yellowish-green to reddish, sparsely velutinous, adaxially yellowish to pinkish-green, inner 2 greenish, elliptic, 6–8 mm long, 3–5 mm wide; androecium sub-actinomorphic, spherical, ca. 5 mm across; stamens 55–90; anthers yellow, slightly compressed, obovate, ca. 1 mm long, apex obtuse, filaments fused at base into a column. **Pistillate flower:** pedicel 1.4–2 cm long, tepals 3, outer 2 suborbicular or broadly ovate, abaxially reddish-green, adaxially yellowish-greenish, 8–12 mm long, 9–13 mm wide, inner 1 yellowish-greenish, elliptic, 5–8 mm long, 2.5–4.5 mm wide; ovary reddish-green, trigonous-ellipsoid, 5–9 mm long, ca. 3 mm thick (wings excluded), sparsely sessile-glandular, 3-winged, wings unequal, reddish-green, lateral wings 2–3 mm wide, abaxial wing crescent-shaped, 3–8 mm wide; styles 3, fused at base, yellow or greenish, *ca.* 4 mm long, stigma spirally twisted. **Capsule** trigonous-ellipsoid, 7–11 mm long, 3–4 mm thick (wings excluded), greenish or reddish when fresh; wings unequal, lateral wings 2–4 mm wide, abaxial wing crescent-shaped, 4–9 mm wide. Somatic chromosome number, 2*n* = 30, 31, 32, 33.

#### Additional specimens examined

VIETNAM, Thanh Hoa Prov., Ba Thuoc Distr., Co Lung Municipality, Eo Dieu village. Primary evergreen seasonal broadleaved and coniferous submontane forest on crystalline marble-like highly eroded limestone, very steep upper slopes and tops of limestone rocky ridge at elev. 800–850 m. Lithophyte. Introduced from home garden plants in Hanoi for cultivation in Academia Sinica. Specimens pressed from greenhouse plants on 1 April 2013, *Ching-I Peng 24610* (HAST); Khuyn village. Primary evergreen seasonal broadleaved lowland forest on crystalline marble-like highly eroded limestone, N slopes of limestone ridge at elev. 400–460 m. Lithophyte on vertical cliff. Occasional. Introduced from home garden plants in Hanoi for cultivation in Academia Sinica. Specimens pressed from greenhouse plants on 22 April 2014, *Ching-I Peng 24613* (HAST).

#### Distribution and ecology

Lithophytic herb on shady cliff. Endemic to Ba Thuoc Dist, Thanh Hoa Province, Northern Vietnam (Figure 3), known only from the type locality. In primary evergreen seasonal broad-leaved closed lowland forest on crystalline marble-like highly eroded limestone, very steep rocky slopes and cliffs at 450–600 m elevation.

#### Etymology

The specific epithet refers to the leaf surface with dense conic bullae, which is reminiscent of the peak clusters in Sino-Vietnamese limestone karst landform.

#### Notes

*Begonia montaniformis* resembles *B. ferox* C.-I Peng & Yan Liu, differing in the stipule margin tomentose (vs. entire), veins silvery (vs. dark green), each bullae 2–4-tipped (vs. 1-tipped) and shortly hispidulous (vs. long velutinous), staminate flower yellowish-green (vs. whitish). The new species is also similar to *B. nahangensis* Aver. & H. Q. Nguyen, another bullate-leaved species recently documented from north Vietnam (Averyanov & Nguyen [2012]), differing in the leaves widely ovate to widely elliptic (vs. broadly oblique-ovate or oblique-reniform), apex caudate (vs. obtuse) and each conic bullae 2–4-tipped (vs. 1-tipped). A detailed comparison of the new species with *B. ferox* and *B. nahangensis* are provided in Table 5.

**Table 5:** **Camparison of** ***Begonia montaniformis*** **with** ***B. ferox*** **and** ***B. nahangensis***

	***B. montaniformis***	***B. ferox***	***B. nahangensis***
**Leaf**			
Bullae	2–4-tipped	Uni-tipped	Uni-tipped
Color	Silvery green along primary and secondary veins against blackish-malachite green background	Embellished dark maroon against green background	Embellished dark maroon against green background
**Inflorescence**			
Peduncle length (cm)	11–26	5–13	(8) 10–12 (14)
**Male flower**			
Tepal color	Yellowish to pinkish-green	Pale pinkish-yellow	White to light pink
**Female flower**			
Tepal color	Yellowish-greenish	Pinkish-white	Light olive-green
Tepal duration	Persistent	Deciduous	Unknown
**Capsule**			
Abaxial wing	Oblique-triangular	Crescent-shaped	Oblique-triangular

### Chromosome cytology

**Figure 14:**
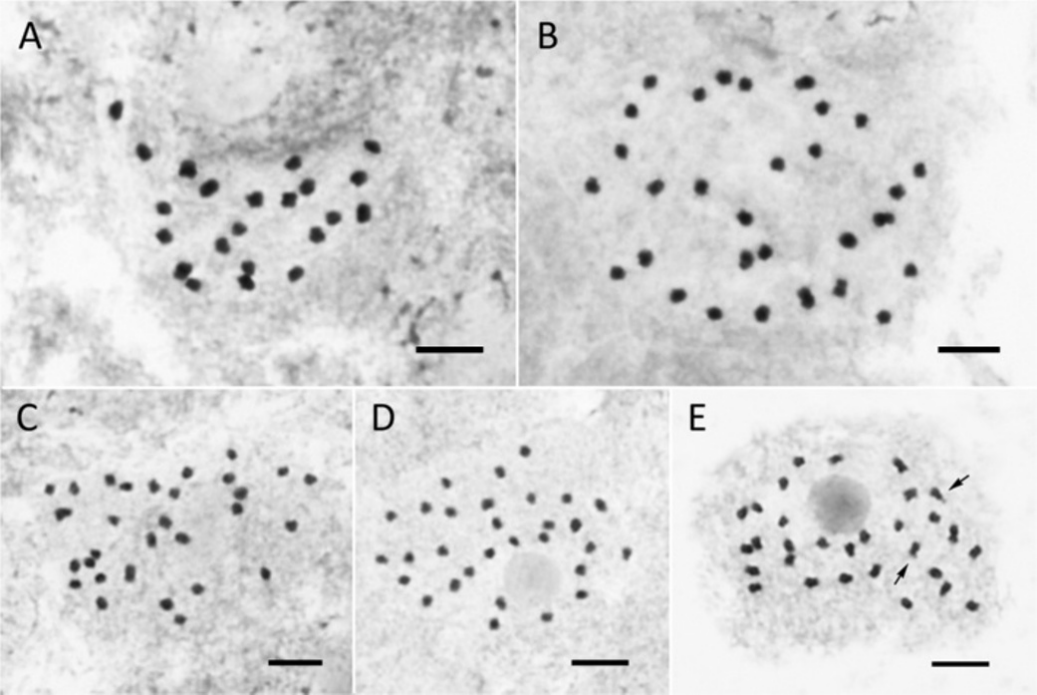
Somatic chromosomes at metaphase of *Begonia*. **A**. *B. caobangensis* (2*n* = 22, *Peng 23895*); **B**. *B. circularis* (2*n* = 30, *Peng et al. 22610*); **C**, *B. melanobullata* (2*n* = 30, *Peng 22610*); **D**. *B. langsonensis* (2*n* = 30, *Peng et al. 21946*); **E**, *B. locii* (2*n* = 30, *Peng et al. 21943*), arrows indicate median chromosomes with satellites. Scale bars = 5 μm.

1. ***Begonia caobangensis*** **(** ***Peng 23895*** **)**, 2*n* = 22, Figure 14A

The 22 chromosomes, ranging from ca. 1.1 to 1.8 μm long, show a gradual change in length. Most chromosomes had centromeres at the median/submedian positions. No satellite chromosomes were observed.

2. ***Begonia circularis*** **(** ***Peng 22610*** **)**, 2*n* = 30, Figure 14B

Among the 30 chromosomes, the first pair of median chromosomes (ca. 2.1 μm long) was relatively longer than the rest and the remaining 28 gradually varied from ca. 1.2 to 1.9 μm long. Except for the shorter chromosomes, most chromosomes had centromeres at the median/submedian positions. No satellite chromosomes were observed.

3. ***Begonia melanobullata*** **(** ***Peng 22609*** **)**, 2*n* = 30, Figure 14C

Among the 30 chromosomes, the first pair of median chromosomes (ca. 1.7 μm long) was longer than the remaining 28 chromosomes (ca. 0.9–1.5 μm). Most chromosomes had centromeres at median/submedian positions. Satellites were not observed.

4. ***Begonia langsonensis*** **(** ***Peng 21946*** **),** 2*n* = 30, Figure 14D

The 30 chromosomes gradually varied from ca. 0.9 to 1.2 μm long. Because of the small chromosome size, centromere positions of chromosomes were difficult to ascertain. Satellites were not observed.

5. ***Begonia locii*** **(** ***Peng 21943*** **)**, 2*n* = 30, Figure 14E

The 30 chromosomes gradually varied from ca. 0.9 to 1.7 μm long. Almost all chromosomes had centromeres at median/submedian positions. Satellites were observed at distal regions of short arms in the second pair of the longest median chromosomes.

**Figure 15:**
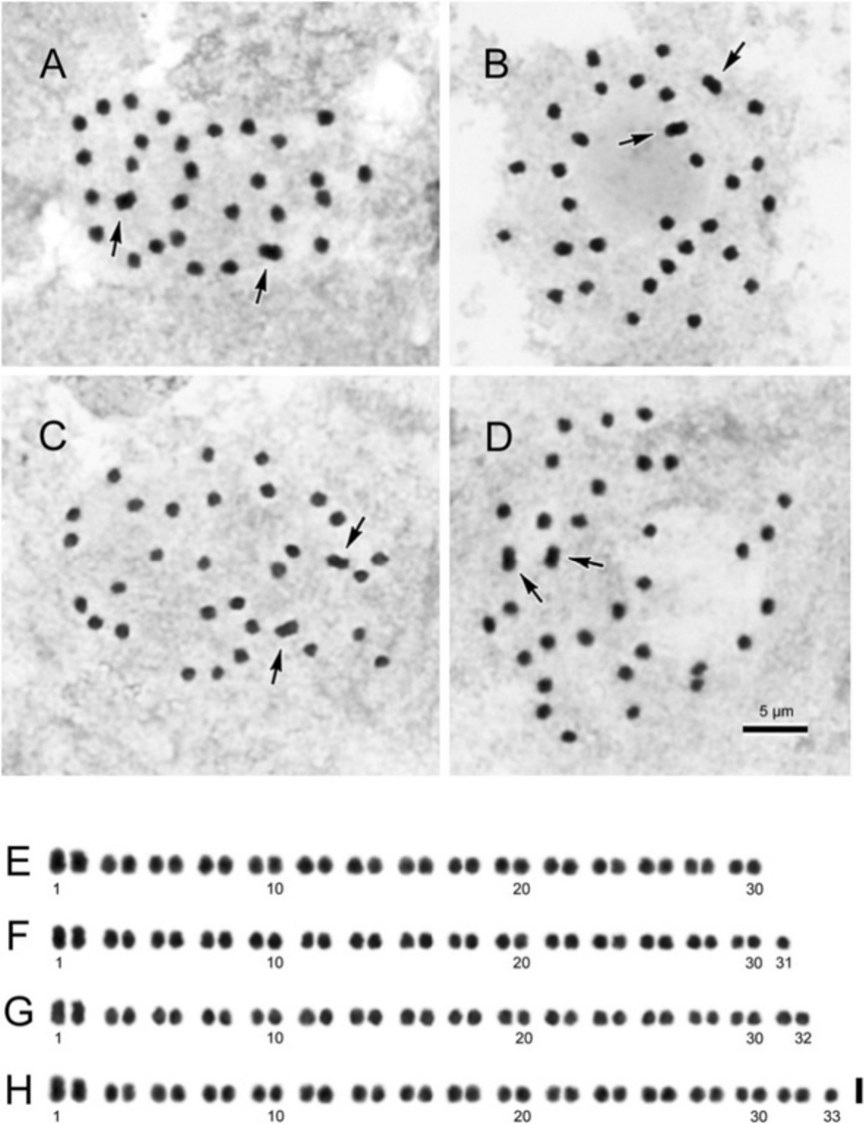
Somatic chromosomes at metaphase of *Begonia montaniformis* (*Peng 24610,* HAST). **A–D**, micrographs; arrows indicate a pair of longer metacentric chromosomes. **E–H**, somatic chromosomes serially arranged by chromosome length and centromere position. **A**, **E**, 2*n* = 30; **B**, **F**, 2*n* = 31; **C**, **G**, 2*n* = 32; **D**, **H**, 2*n* = 33. Scale bars = 5 μm **(A–D)**; 2 μm **(E–H)**.

6. ***Begonia montaniformis*** **(** ***Peng 24609, Peng24610, Peng24613*** **)**, 2*n* = 30, 31, 32, 33, Figure 15

The holotype (*Peng 24609*) and one of the paratypes (*Peng 24613*) had 2*n* = 30, which is the prevalent chromosome number for members of sect. *Coelocentrum*. However, another collection (*Peng 24610*) showed aneuploidy with 2*n* = 30, 31, 32, 33 in different individuals. The aneuploids shared chromosomal features in common in the respective chromosome complements: The first two chromosomes were relatively longer, about 1.8–2.0 μm (arrows in Figures A-D; Nos. 1, 2 in Figures E-H), and the rest were shorter, about 1.0-1.5 μm long. The two longest chromosomes were clearly metacentric, however, the centromere positions of most other chromosomes could not be determined. Satellites were not observed.

To sum up, the chromosome number 2*n* = 22 for *B. caobangensis* agrees with those of the majority species delimited in Asian section *Platycentrum* (Y. Kono and C.-I Peng, unpublished data). The chromosome count of 2*n* = 30 in four species (*Begonia circularis*, *B. melanobullata*, *B. langsonensis*, *B. locii*) of sect. *Coelocentrum* is consistent with previous studies on members in this section (Peng et al. [2013], [2014a]). Unexpected aneuploid chromosome numbers (2*n* = 30, 31, 32, 33) however were observed in *B. montaniformis*. A review of literature revealed that aneuploidy was documented in several species of *Begonia*, e.g. *B. formosana* (2*n* = 60, 64: Oginuma and Peng, [2002]); *B. longipetiolata* (2*n* = 34, 36, 38, 71–73: Arends, [1992]).

### Leaf anatomy and vestiture

1. ***Begonia caobangensis***

**Figure 16:**
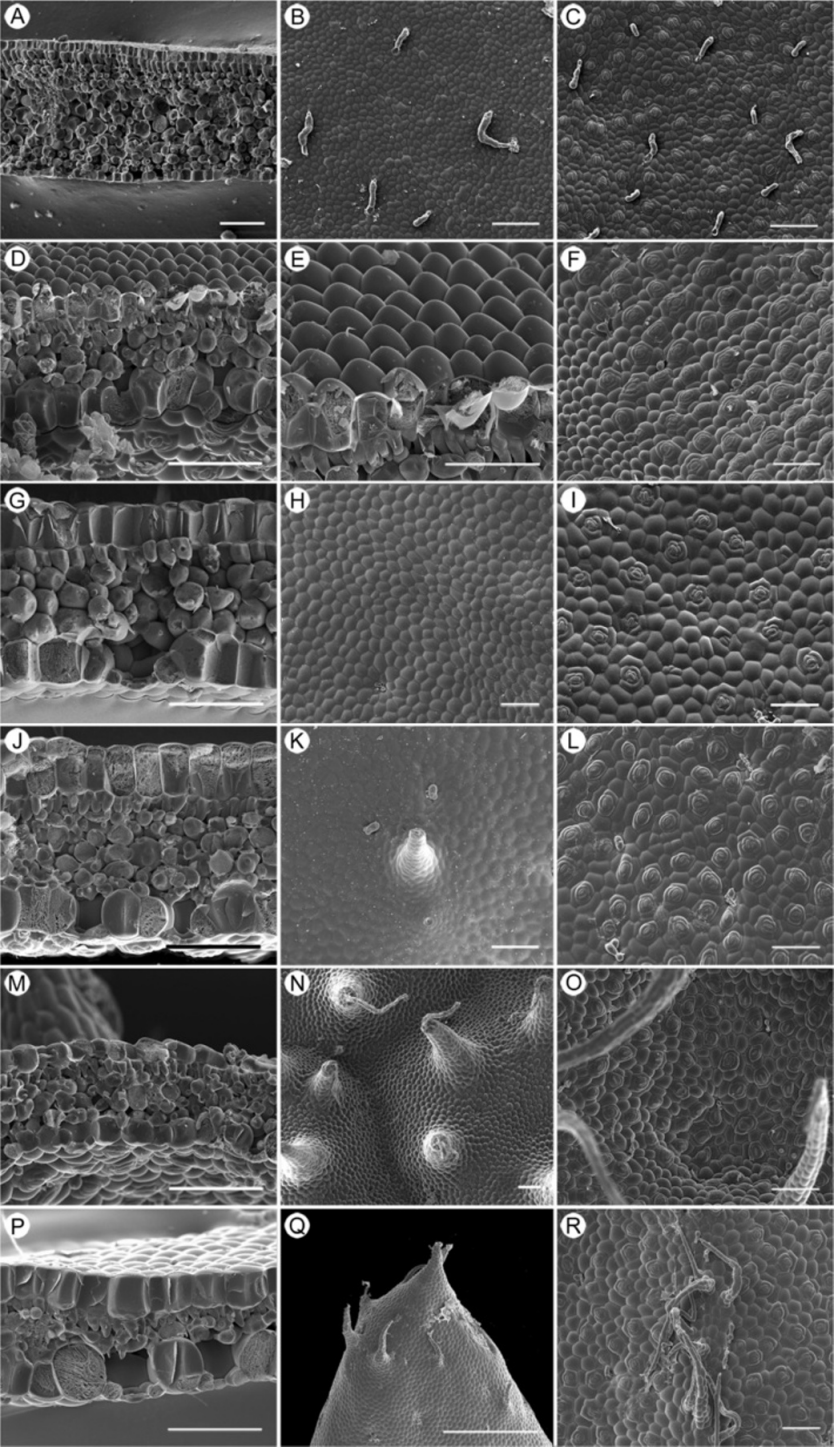
Leaf SEM microphotographs of *Begonia*. **A–C**, *Begonia caobangensis*; **D–F**, *B. circularis*; **G–I**, *B. melanobullata*; **J–L**, *B. langsonensis*; **M–O**, *B. locii*; **P–R**, *B. montaniformis.* **A**, **D**, **G**, **J**, **M**, **P**, Cross section; **B**, **E**, **H**, **K**, **N**, **Q**, Adaxial surface; **C**, **F**, **I**, **L**, **O**, **R**, Abaxial surface. (Scales = 200 μm).

Cross section ca. 550 μm thick; epidermis single-layered on both surfaces, hypodermis absent; palisade parenchyma cells 1-layered; spongy parenchyma cells ca. 10-layered (Figure 16A); adaxial and abaxial surfaces with glandular trichomes (Figures 16B,C); stomata complex single, helicocytic, flat, subsidiary cells 3 (Figure 16C).

2. ***Begonia circularis***

Cross section ca. 250 μm thick; epidermis single-layered on both surfaces, hypodermis absent; palisade parenchyma cells 1-layered; spongy parenchyma cells ca. 3-layered (Figure 16D); adaxial surface conoidal (Figure 16E) like those of *B. pengii* (Ku et al. [2008]: figure six C); abaxial surface with sparse glandular trichomes (Figure 16F); stomata complex single, helicocytic, slightly elevated, subsidiary cell 5–6 (Figure 16F).

3. ***Begonia melanobullata***

Cross section ca. 390 μm thick, epidermis single-layered, hypodermis absent; palisade parenchyma cells 1-layered; spongy parenchyma cells ca. 3-layered (Figure 16G); both surfaces with sparse glandular trichomes (Figures 16H,I); stomata complex single, helicocytic, nearly flat, subsidiary cell 3–7 (Figure 16I).

4. ***Begonia langsonensis***

Cross section ca. 420 μm thick, epidermis single-layered, hypodermis absent; palisade parenchyma cells 1-layered; spongy parenchyma cells ca. 5-layered (Figure 16J); both surfaces with glandular trichomes (Figures 16K,L); stomata complex single, helicocytic, nearly flat, subsidiary cell 5 (Figure 16L).

5. ***Begonia locii***

Cross section ca. 200 μm thick; epidermis single-layered, hypodermis absent; palisade parenchyma cells 1-layered; spongy parenchyma cells ca. 4-layered (Figure 16M); adaxial surface slightly conoidal and with multiseriate trichomes (Figure 16N), both surfaces with sparse glandular trichomes (Figures 16N,O); stomata complex single, helicocytic, slightly elevated, subsidiary cell 4–5 (Figure 16O).

6. ***Begonia montaniformis***

Cross section ca. 280 μm thick, epidermis single-layered, hypodermis absent; palisade parenchyma cells 1-layered; spongy parenchyma cells ca. 2-layered (Figure 16P); adaxial surface conical-bullate, each bullate with several multiseriate trichomes near the tip, abaxial surface with multiseriate trichomes on veins, both surfaces with sparse glandular trichomes (Figure 16Q,R); stomata complex single, helicocytic, nearly flat, subsidiary cell 5 (Figure 16R).

## Conclusion

A careful study of the literature, herbarium specimens and living plants, both in the wild and in cultivation in the experimental greenhouse, supports the recognition of the six new species, which are described and illustrated. Our study also suggests that there are more new species in limestone karst areas across the Sino-Vietnamese border region to be discovered in the future.

## Authors’ original submitted files for images

Below are the links to the authors’ original submitted files for images. Authors’ original file for figure 1 Authors’ original file for figure 2 Authors’ original file for figure 3 Authors’ original file for figure 4 Authors’ original file for figure 5 Authors’ original file for figure 6 Authors’ original file for figure 7 Authors’ original file for figure 8 Authors’ original file for figure 9 Authors’ original file for figure 10 Authors’ original file for figure 11 Authors’ original file for figure 12 Authors’ original file for figure 13 Authors’ original file for figure 14 Authors’ original file for figure 15 Authors’ original file for figure 16
